# Seaweed as a climate fix for meat and dairy production: an LCA perspective

**DOI:** 10.1038/s41598-025-18322-1

**Published:** 2025-09-18

**Authors:** J.-B. E. Thomas, V. W. Xu, S. J. Krizsan, I. M. Aasen, A. Oliveira, H. Ramos, P. Tyedmers, M. Hayes, F. Gröndahl

**Affiliations:** 1 Department of Sustainable Development, Environmental Science and Engineering, KTH Royal Institute of Technology, Teknikringen 10B, 100 44 Stockholm, Sweden; 2 Department of Agricultural Sciences, Faculty of Applied Ecology, Agricultural Sciences and Biotechnology, Inland Norway University of Applied Sciences, Campus Blæstad, 2322 Hamar, Norway; 3 Department of Biotechnology and Nanomedicine, Industrial and Marine Biotechnology, SINTEF Industry, 7465 Trondheim, Norway; 4 Seaexpert, Ltd., Travessa Do Farrobim 15, 9900-361 Horta, Faial, Azores Portugal; 5 School for Resource and Environmental Studies, Dalhousie University, 6100 University Ave, PO Box 15000, Halifax, NS Canada; 6 Food BioSciences Department, Teagasc Food Research Centre, Ashtown, Dublin 15, Ireland

**Keywords:** Environmental impact, Animal biotechnology

## Abstract

Livestock supply chains contribute a substantial share of global anthropogenic greenhouse gas (GHG) emissions, with enteric methane (CH₄) from ruminants being a key driver. Seaweed-derived feed additives have been proposed as a CH₄ mitigation strategy, but their broader environmental trade-offs remain unclear. This study applies life cycle assessment (LCA) to evaluate seven seaweed-supplemented scenarios across beef, dairy, and sheep production, assessing climate change, marine and freshwater eutrophication, land use, fossil fuel depletion, and water use. Results indicate that while certain seaweed additives can lower CH₄ emissions in vitro, real-world reductions in total GHG emissions remain modest. Energy-intensive processing and long transport distances can offset CH₄ abatement gains, with only scenarios utilizing low-impact by-products achieving net climate benefits. Sensitivity analyses highlight the importance of Global Warming Potential (GWP) time horizon selection, energy sources, and grazing practices in determining overall environmental performance. Optimizing algae sourcing, processing, and application will be essential to realizing meaningful and scalable mitigation potential in ruminant systems. While seaweed additives can contribute to CH₄ reduction, this study concludes they will not single-handedly deliver transformative climate benefits.

**Supplementary Information:**

The online version contains supplementary material available at 10.1038/s41598-025-18322-1.

## Introduction

Livestock supply chains are amongst the single most important sources of emissions driving human-induced climate change, accounting for an estimated 6.2 gigatons of CO_2_-equivalent emissions per annum, or approximately 12% of all global anthropogenic greenhouse gas (GHG) emissions^1^. Without interventions, emissions are expected to rise to 9.1 gigatons by 2050, largely driven by increased global demand for livestock products^1^. Livestock production systems are therefore a strategic priority in global efforts to reduce GHG emissions and meet international climate targets such as the Paris Agreement^2^. Key GHG emission hotspots across the full life cycle of livestock production vary between species and from one production setting to the next. However, they typically include feed production and associated land-use change particularly when recent deforestation has, or is, occurring for pasture and feed crops, along with manure management, N_2_O from dung and urine in paddocks^3^, and enteric methane (CH_4_) emissions from ruminants like cows and sheep^4,5^. Of the three major GHGs that arise from these hotspots—CO_2_, N_2_O and CH_4_, it is CH_4_ that accounts for approximately half of the atmospheric loading from livestock production up to farmgate, with enteric fermentation accounting for the majority of these emissions^6–8^. As such, enteric CH_4_ emissions from ruminants could be responsible for 5 to 6% of global anthropogenic GHG emissions.

A wide range of strategies to reduce enteric methane emissions in ruminants are currently under investigation, including dietary interventions such as fuzzy cottonseed, monensin, and halogenated compounds^9^. Research on halogenated compounds has evolved over several decades, with the earliest studies traceable to the 1960s. In the early 2000s, bromochloromethane (BCM) was identified as an effective anti-methanogenic additive, demonstrating methane reductions of up to 93% in cattle^10^. However, due to concerns over potential residues and environmental safety, BCM was subsequently banned in Australia, limiting its commercial application. This historical precedent shaped interest in natural alternatives, particularly the red seaweed *Asparagopsis taxiformis*, which contains bromoform (CHBr₃) as its active methane-suppressing compound. Machado, Magnusson^11^ demonstrated CH_4_ reductions of over 90% from rumen fluid sourced from cattle using the red seaweed *Asparagopsis taxiformis* under controlled conditions, attributed to the bioactive bromoform compound CHBr₃. This sparked further interest to confirm these original in vitro results^12–14^, leading to studies on in vivo CH_4_ reduction potential of *Asparagopsis spp.*, testing efficacy amongst dairy cows^15,16^, beef cattle^17,18^, and sheep^19^. The use of different biomass processing methods was also identified as resulting in variations in elemental, secondary metabolite and nutritional composition, which in turn also affects potential anti-methanogenic^20,21^. In addition to CH_4_ reduction, co-benefits such as increased weight gain have been reported^17^. However, given the structural similarity of CHBr₃ to BCM, concerns persist regarding its long-term effects on livestock health, regulatory approval, and environmental impact. Bromoform emissions from seaweeds have been identified as a significant contributor to atmospheric halocarbon fluxes^22^, with potential implications for ozone depletion. While *Asparagopsis* remains a promising CH_4_ mitigation strategy, further research is needed to assess its sustainability and regulatory feasibility.

Although most of the focus of this research has been on *Asparagopsis* spp., a few other studies have also explored the CH_4_ emission reduction potential of other seaweed genus and species, particularly those found in the North Atlantic^23–27^. The study of Nunes, Maduro-Dias^26^ is one of the most recent, in which they evaluated the potential of *Rugulopteryx okamurae*, an invasive brown macroalgae that is severely affecting the marine coastal biodiversity in the Mediterranean Sea (south of Spain, Portugal and North of Africa) and Macaronesia, for incorporation into cattle feed as an anti-methanogenic agent. Results from that in vitro study showed that the inclusion of 1% and 5% dry matter of *R. okamurae* in the cattle diet resulted respectively in a 38% and 98% decrease (*p* < 0.05) in CH_4_ production after 24h of incubation, although it also resulted in a significant decrease (*p* < 0.05) in gas production of 57.02% and 73.5% with the inclusion of 1% and 5% seaweed additive, respectively, during 96 h.

There are also studies exploring this subject on the Pacific coast, as shown by^14^ in which they targeted the local Californian coast sourced *Asparagopsis taxiformis* and *Zonaria farlowii* to study their ability to mitigate CH_4_ production in dairy cattle when added to its feed. These in vitro studies showed a reduction in CH_4_ production by up to 74% (p < 0.05) using *A. taxiformis* and 11% (*p* < 0.05) using *Z. farlowii* during rumen fermentation, with no effect on CO_2_ production in either case. All in vitro studies performed anywhere always reinforced the need to follow-up the work with in vivo studies in order to confirm prior enteric CH_4_ reduction values, but also for biomass toxicity and animal health assessment^14,24,26^.

Ireland has one of the most established seaweed industries in Europe and is the fourth largest EU player in seaweed processing. In 2018, 77,000 tonnes of seaweed worth €37 million were exported and 58,000 tonnes (worth €9 million) were imported for reprocessing and export markets^28^. Each year approximately 30,000 wt/yr of the brown seaweed *Ascophyllum nodosum* is wild harvested and processed for use mainly as a starter material for biostimulant manufacture, as an animal feed additive or as a source of fucoidan^29^. A study performed by Roskam and colleagues, using *A. nodosum* extract (EX1) in mature ewes over two time periods of eight weeks, found that EX1 reduced CH_4_ g/d and CH_4_ g/kg Dry Matter Intake (DMI) (*p* < 0.05) by 9% and 10%, respectively^30^. In addition, a further study looked at the potential of dietary supplementation of *A. nodosum* or treated *A. nodosum* in an intensive beef cattle feeding system on animal performance, gaseous emissions, ruminal fermentation and microbiota, and muscle fatty acid profiles. Seventy-two dairy-beef bulls (380 kg; 11 months of age) were randomly allocated to one of four dietary treatments (n = 18) for a 70-d period. *A. nodosum* and *A. nodosum* extract reduced CH_4_ g/day (d) (*p* < 0.001), CH_4_ g/kg DMI, (*p* < 0.01), and CH_4_ g/kg Body weight (BW), (*p* < 0.001) and the extract reduced CH_4_ g/d by 8% (*p* < 0.001) and tended to reduce CH_4_ g/kg BW by 7% (*p* = 0.07)^31^.

An interesting alternative to harvesting or cultivation of biomass just for the purpose of CH_4_ mitigation, is the valorisation of waste and co-products generated during the extraction of food or hydrocolloid ingredients from seaweeds. In Europe (Norway and France), 250 000 tonnes wet weight of *Laminaria hyperborea* and *Laminaria digitata* are harvested annually for alginate production^32,33^. This production generates a residue constituting 20–25% of the dry weight, or 6–8 000 tonnes. This residue is currently a waste product going to landfill or returned to the sea. The methane mitigation potential of this co-product residue was tested in a sheep feeding trial^34^. A 25% reduction of CH_4_ emissions and CH_4_ yield was observed after the first five weeks of the trial, while the reduction over the total experimental period of 13 weeks was less than 10% and not significant due to large individual differences between the animals.

As of December 2024, only two Life Cycle Assessment (LCA) studies have been reported in the literature that evaluate the production impacts and the potential emission reduction benefits of seaweed feed supplements in ruminant diets. The first focused on the land-based production of dried *Asparagopsis taxiformis* in part to address concerns about the need to produce large quantities of *Asparagopsis* spp. at low cost to make a significant global CH_4_ reduction contribution. These authors found that 1kg of dried product (post-processing gate) resulted in 9.2 kg CO_2_e of Global Warming Potential (GWP100), though optimisation scenarios of the production system using sea salt and heat pumps, found production related emissions could be as low as 5.8 and 8.4 kg CO_2_e per kg dry product, respectively^35^. Building on this study, a follow-up LCA explored the GHG emissions reduction potential of feeding *Asparagopsis taxiformis* to dairy cows in Germany under both a high (191 g/day) and low (95.5 g/day) algal supplementation regimen. In this work, Méité and colleagues, found rumen-sourced CH₄ reductions of 65.5% and 29.4% for the high and low intake scenarios, respectively. However, when combined with all other sources of life cycle GHG emissions associated with the feeding and management of this herd per unit of milk produced, overall emissions reductions amounted to 23% and 10% respectively relative to milk produced without any algal dietary supplementation^36^. These findings thus highlight that a reduction of two thirds of enteric CH_4_ emissions corresponds to a reduction of approximately one quarter of the total climate impact of milk production.

A recent study by Ridoutt, Lehnert^37^ sought to estimate the GHG emission reduction benefits of incorporating *Asparagopsis taxiformis* into beef cattle diets in feedlots in Australia. Rather than following ISO-compliant LCA methods, the study adopted a scenario-based GHG modelling approach with a life cycle perspective. Using secondary data drawn from the literature and modelled emissions, the study explored seven adoption scenarios, however it focuses only on the feedlot stage rather than a full life cycle perspective that include pasture-based systems before feedlot entry. Based on their modelling, Ridoutt and colleagues predicted enteric fermentation related CH₄ reductions of up to 98% during finishing and if widely adopted across feedlots, overall GHG emissions reductions of 1–4% for the beef sector by 2030. This modest sector-wide reduction reflects the fact that CH₄ emissions, while the dominant GHG from enteric fermentation, make up only a portion of the beef sector’s total GHG emissions, with CO₂ and N₂O from land use, feed production, and CH_4_ from manure management also contributing significantly. While focusing narrowly on GHG impacts and lacking a detailed inventory or system boundary definition, the study suggests potential reductions in the feedlot sector’s radiative forcing, potentially achieving climate neutrality. However, this falls short of the broader environmental impact insights offered by ISO-standard LCAs.

While significant progress has been made in understanding the biochemical mechanisms underlying CH_4_ reductions from seaweed feed supplements, as well as identifying promising alternatives to *Asparagopsis taxiformis*, such as *Ascophyllum nodosum*, and *Laminaria hyperborea*, the environmental implications of scaling up these solutions and fully accounting for both known and novel sources of inputs and impacts remain underexplored. Only a few studies have applied LCA methods to evaluate the potential climate impacts of integrating one or another seaweed into one or another ruminant diet. Here, we expand on this foundation, using LCA methods to investigate not only the climate impact reduction potential of introducing seaweed-derived additives from multiple species into ruminant diets used to produce both milk and meat, but also how supplementation may affect other key global scale environmental concerns. By assessing impacts across a suite of impact categories, and including production, processing, and implementation of algal supplements into ruminant diets, this study contributes to a better understanding of the trade-offs and opportunities associated with these enteric CH_4_ mitigation strategies.

## Material and methods

LCA is an established analytical framework designed to evaluate the resource depletion and environmental impact contributions associated with a product across its entire life cycle. This approach encompasses all stages, from raw material extraction and processing, through production and distribution, to usage and eventual end-of-life treatment. Conducted in accordance with the standardized methodologies prescribed by ISO14040 and ISO14044, LCA is structured into four principal phases. First, the Goal and Scope Definition phase identifies the purpose of the study and delineates the system boundaries. Second, the Life Cycle Inventory (LCI) Analysis involves systematically gathering and quantifying data on resource inputs, energy flows, and emissions. Third, the Life Cycle Impact Assessment (LCIA) phase uses peer-reviewed models that translates the LCI data into contributions to environmental impact categories of concern in a given study to evaluate potential effects. Finally, the Interpretation phase synthesizes the findings, enabling the development of conclusions and actionable recommendations. This structured methodology ensures a robust and transparent analysis of environmental performance.

### Goal and scope definition

**Fig. 1:**
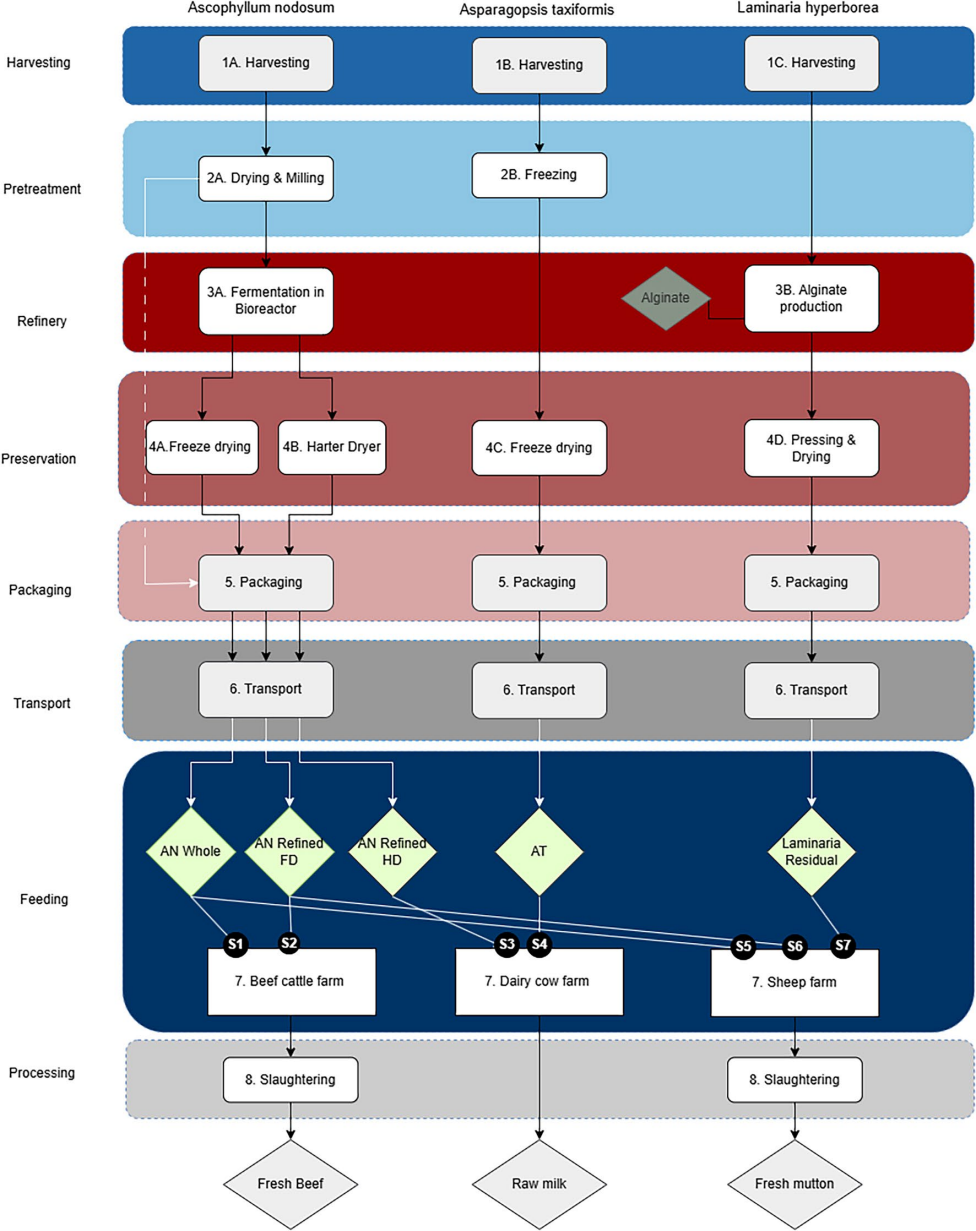
Systems diagram. Stocks are represented by diamond shapes, flows by arrows and processes by boxes.

The primary objective of this LCA is to evaluate the environmental impacts of incorporating various seaweeds as feed additives into diets of cattle and sheep to reduce the GHG emissions intensity of livestock products destined for human consumption (i.e. milk and edible bone-free meat). Specifically, the study evaluates the use of *Ascophyllum nodosum, Laminaria hyperborea,* and *Asparagopsis taxiformis* added to diets at specific rates to produce milk from dairy cows, beef from beef cattle, and mutton from sheep in typical European systems. A total of seven scenarios are assessed relative to reference baseline scenarios of the milk, beef and mutton being produced *without* seaweed feed additives (see Fig. 1). This research aims to inform industry stakeholders and the scientific community about the realistic GHG emissions reduction potential of seaweeds in livestock diets, thereby contributing to academic knowledge and potentially guiding future industry practices. Though algal supplementation of ruminant diets is being promoted expressly to limit life cycle GHG emissions associated with the production of milk and meat from these animals, as provision of these novel feed inputs will contribute to a wider range of resource depletion and environmental concerns, we also report on likely changes in marine and freshwater eutrophication, land use, water use, and resource depletion (fossil fuels). Furthermore, CH₄ is a GHG with a relatively short atmospheric lifespan, typically breaking down within 12 years under standard conditions^38^, therefore the modelling of Global Warming Potential (GWP) was subject to sensitivity analysis in the final interpretation of results, comparing the Environmental Footprint (EF) standard GWP_100_ with GWP_20_ and GWP_500_. Unless otherwise specified, GWP will refer to GWP_100_ throughout this study.

In each of the seven models (referred to as scenarios), the functional unit is one kilogram of human edible product, defined as 1L of raw milk, or 1kg of bone-free beef or mutton, depending on the scenario. Note that 1L of raw milk is considered equivalent to 1kg in this study. This cradle-to-gate LCA includes all processes from raw material extraction to the slaughterhouse gate for beef and mutton or to post-milking farm-gate for dairy. For a full description of the farm systems, see Sects. "Beef production system", "Raw milk production system" And "Mutton production system" for beef, raw milk and mutton production, respectively. At slaughter, environmental burdens are partitioned among boneless meat (the functional unit) and all other co-products (hides, edible offal, feed-grade fractions, etc.) in proportion to their mass specified in Sects. "Beef production system", "Raw milk production system" and "Mutton production system". Only the share assigned to boneless meat is included in our results; impacts allocated to the other fractions are excluded from further analysis.

The extent of pasture grazing in livestock systems varies geographically; for example, sheep, cattle, and dairy cows in Ireland may be moved indoors for winter later than in Scandinavia, influencing the type and quantity of feed consumed. The extent of grazing was therefore subject to sensitivity analysis. This study does not consider grazing quality across the cases, though this could be an important factor affecting land use change, ruminant emissions and feed requirements. Similarly, grazing would likely also affect the standard edible yield, however given a lack of observable data and for the sake of simplicity, this was assumed to remain constant regardless of the extent of grazing dependence. The study models a replacement of approximately 2–4% (depending on the scenario) of the feed consumed by animals with the seaweed additives relative to baseline diets, and is assumed to be replacing feed year-round, even when animals are on pasture. The system boundary includes seaweed cultivation or harvesting, processing, feed production, livestock rearing, and associated emissions. The inventory data for the seaweed and additive production is based on current, small scale production systems as described in Sects. "Ascophyllum nodosum (Linnaeus) Le Jolis", "Asparagopsis taxiformis (Delile) Trevisan 1845" and "Laminaria hyperborea (Gunnerus) Foslie 1885".

The project did not directly measure GHG emission reductions in the manure management stage, which can also result from seaweed additives, though previous research suggests these would be relatively small compared to enteric CH_4_ emission reductions^36^. Transport processes are modelled based on actual routes and modes of transport used in contemporary algal supply chains that were used to characterize inputs to the production and processing of the diet supplements. As these distances do not represent optimized value chains, transport distances are also subject to sensitivity analysis.

The European Commission endorsed EF3.1 method was selected for the LCIA phase for its comprehensive, standardized approach aligned with international LCA best practices, ensuring consistent and transparent impact assessment^39^. To enhance the relevance and manageability of our analysis, we focused on six key impact categories: climate change, marine and freshwater eutrophication, land use, water use, and resource depletion (fossil fuels). This targeted selection was driven by their significant relevance to the life cycle of livestock products. Climate change and energy use highlight carbon footprint and efficiency, while eutrophication reflects major ecological pressures. Land and water use were included as indicators of resource sustainability, offering insights into spatial and hydrological impacts. Focusing on these categories improves data efficiency and enhances result interpretation for stakeholder engagement and decision-making. Climate change is highlighted in the main text because enteric-methane abatement is the explicit policy driver behind algae-feed research. To guard against the potential for environmental burden-shifting criticised by Brock and Tan^40^, we nevertheless quantify, and later discuss, five additional EF-3.1 categories (marine and freshwater eutrophication, land use, water use, and fossil-fuel depletion).

### Systems description

The seven scenarios (S1-S7) depicted in the present study are direct representations of the flows that occurred in the course of the studies by^34^, De Bhowmick, Rai^41^ and Roskam, O’Donnell^30^. Not all seaweed additives were in vitro tested for each livestock type, and enteric CH₄ reduction values are based on in vivo results from the three aforementioned studies. Table 1 provides an overview of each product system scenario, including seaweed species, processing method to produce the additive, and the associated product system (beef, mutton or dairy). Figure 1 is a systems diagram providing an overview of each product system’s scenarios and the processes that constitute each system’s life cycle stages. The beef product systems models a self-replicating Irish beef cattle system (≈53 °N, 8 °W) in which calves graze on pasture 58.6% of the year and are finished indoors on grass silage and compound feed to 647 kg live weight at 2 years. The dairy system represents a temperate-climate, mixed breed dairy farm (≈52 °N, 5 °E) based on Agri-footprint^42^, combining on-farm forage cultivation and pasture management with economic allocation across milk and culled animal coproducts. The mutton system reflects average U.S. sheep farming (≈39°N, 98°W) across 80% extensive and 20% intensive pasture systems, using the allocation at point of substitution (APOS) allocation approach at the farm gate for meat and unwashed wool outputs. This system was selected due to the availability of disaggregated enteric methane emission data, which is critical for assessing CH₄ mitigation, and because its management practices are sufficiently representative of Irish sheep systems, for which enteric CH₄ emissions were not reported separately. All systems are described in detail below in Sects. "Beef Production System"-"Laminaria hyperborea (Gunnerus) Foslie 1885".

**Table 1:** Overview of the seven scenarios.

System	Scenario	Algal species	Treatment	Supplementation rate	Functional unit
Cattle	S1	*Ascophyllum nodosum* (AN)	AN whole (dried and milled into powder)	140 g per cattle per day	1 kg boneless beef
	S2	*Ascophyllum nodosum* (AN)	AN refined (processed and freeze-dried)	140 g per cattle per day	1 kg boneless beef
Dairy Cows	S3	*Ascophyllum nodosum* (AN)	AN refined (processed and harter-dried)	0.5% of organic matter per day	1 L raw milk
	S4	*Asparagopsis taxiformis* (AT)	AT dried (freeze-dried)	0.5% of organic matter per day	1 L raw milk
Sheep	S5	*Ascophyllum nodosum* (AN)	AN whole (dried and milled into powder)	30 g per sheep per day	1 kg boneless mutton
	S6	*Ascophyllum nodosum* (AN)	AN refined (processed and freeze-dried)	60 g per sheep per day	1 kg boneless mutton
	S7	*Laminaria hyperborea* (LH)	LH residual (residue from alginate extraction)	30 g per sheep per day	1 kg boneless mutton

#### Beef production system

Dedicated beef production systems vary widely across the world and across several factors, including breed of animal, the age and weight of the animals at the time of slaughter, the type of housing system (indoor, outdoor, or a combination) used to finish the animals, and the components of the feed. Though each of these factors can affect the life cycle environmental performance of a beef system to one extent or another, for modelling simplicity and reproducibility, in this study, the baseline system ‘Beef cattle for slaughter, at beef farm/IE Mass’ by Blonk Agri-footprint BV is used. The system describes a self-replicating Irish beef system for beef production, including a bull kept on pasture. In this system, beef calves are primarily fed on grass in pasture for 58.6% of the year (214 days), and on grass silage and compound feed in stable for 41.4% of the year (151 days). Calves are weaned after roughly 6 months and the meat calves are slaughtered at two years of age. The target weight of meat calves is 647 kg. For meat production, three cows and fifteen 2-year old calves are slaughtered every year. This Irish beef system is based on a study by Casey and Holden^43^ but has been adapted to reflect the production system case data available in the project, first by direct substitution of feed for seaweed ingredient, and second by adjusting the enteric methane emissions based on project data (specified in Sect. "Ascophyllum nodosum (Linnaeus) Le Jolis" below). The 647kg of live beef produces 296.3kg of beef meat fresh (45.8%), 120.9kg food grade by-product (18.7%), 91.2kg feed grade co-product (14.1%), 138.5kg other co-products (21.4%).

The total GWP of the system is 17.04 kg CO_2_e per kg edible beef (see Supplementary Fig. 1), which is a relatively low climate impact for beef product systems^44^ but in-line with expectations of Irish beef production^45^. In the life cycle of beef cattle farming, the beef fattening phase accounts for approximately 41% of total GWP at 6.98 kg CO_2_e. Other significant contributors include compound feed at 3.84 kg CO_2_e, grass in pasture at 2.82 kg CO_2_e, and grass silage at 2.31 kg CO_2_e. Diesel burned in machinery and electricity consumption contribute smaller amounts, with values of 0.54 and 0.27 kg CO_2_e, respectively. Transport has the least impact, accounting for only 0.02 kg CO_2_e.

#### Raw milk production system

The cow milk production system, modelled in SimaPro 8 using Agri-footprint data, consists of a mixed herd of 100 dairy cows (69% Holstein Friesian, 13% Jersey, 5% Red, and 13% Crossbreeds/others) and 102 replacement animals. The system integrates both livestock and forage cultivation, with roughage production and pasture management occurring on the farm. Calves are either sold for slaughter or raised as replacements, while culled dairy cows are treated as co-products of the system. The dataset applies economic allocation, with inventories covering crop cultivation, animal production, and product processing^46^.

Farm inputs encompass fertilizers, pesticides, irrigation water, lime, diesel, electricity, and capital goods. The environmental impact of drained organic soils and direct Land Use Change (LUC) for maize silage is accounted for, while additions or loss of soil organic carbon from pastured grassland is assumed to be zero. Key emissions assessed include N_2_O, NH_3_, and NOx from crop residues and fertilizers, nitrate and phosphate leaching, CO_2_ from lime and urea application, and heavy metals released into water and soil^46^. The system’s main outputs include culled cows (29.5 animals at 653 kg liveweight), calves (26.8 animals at 45 kg), and heifers (14.3 animals at 555 kg). Additionally, raw milk production amounts to 1,059,335 kg annually, with a fat-corrected composition of 4% fat and 3.3% protein^46^.

The GWP of dairy cow farming is distributed across several contributing factors, including electricity use, livestock categories, and other farm-related emissions (see Supplementary Fig. 2). The total GHG emissions of the system is modelled 1.038 kg CO_2_e/L. Among the various contributors, CH_4_ emissions from dairy cows are the largest source of GHGs, accounting for 0.388 kg CO_2_e. This is followed closely by other miscellaneous emissions, which contribute 0.382 kg CO_2_e. Calves also represent a significant portion, with the emission of 0.235 kg CO_2_e, while heifers account for a smaller share of 0.024 kg CO_2_e. Electricity use contributes the least, with an impact of just 0.010 kg CO_2_e. This data highlights the significant role of CH_4_ and livestock-related emissions in the overall environmental footprint of dairy farming.

#### Mutton production system

The sheep production dataset used as the basis to model Irish mutton production provides an overview of average U.S. sheep farming practices for meat and raw unwashed wool production under conventional conditions. This system represents the husbandry of one sheep over one year, with outputs including 4.2 kg of sheep fleece in grease and 62.8 kg of sheep live weight for slaughter, which in turn results in 28.8 kg meat and 34 kg of carcass. The production is based on data extrapolated from U.S. sources, literature, farm surveys, and expert knowledge. It accounts for inputs such as feedstuffs, fertilizers, pesticides, and irrigation on 80% extensive and 20% intensive pasture systems. The dataset also includes infrastructure like machine sheltering sheds and field emissions, while excluding processes beyond the farm gate (e.g., transport, slaughtering, and cooling).

This dataset was initially included in Ecoinvent version 2 and updated to version 3 with adjustments for consistent water flows and improved documentation. It adheres to Ecoinvent’s quality guidelines and captures the environmental and resource expenditures of sheep farming up to the farm gate. The total production system described applies the APOS modelling approach.

The life cycle stages of mutton farming contribute to GWP through various activities, including feed production, CH_4_ emissions, and infrastructure use (see Supplementary Fig. 3). The total GWP of the system is 5.806 kg CO_2_e/kg bone free meat. Among the contributors, CH4 emissions are the largest source, accounting for 3.511 kg CO_2_e, or approximately 60.47% of the total. Feed production represents the second largest impact, contributing 1.059 kg CO_2_e. Other sources of emissions, categorized as miscellaneous, account for 0.890 kg CO_2_e, followed by fertilizing activities with 0.182 kg CO_2_e. The shed infrastructure contributes 0.132 kg CO_2_e, while slaughtering activities add a smaller portion of 0.028 kg CO_2_e. Irrigation has the least impact, contributing just 0.005 kg CO_2_e. These data emphasize the dominant role of CH_4_ emissions and feed production in the environmental footprint of mutton farming. Compared to beef farming, the sheep fattening phase contributes to a larger share (72.3%) of total GWP.

#### Ascophyllum nodosum (Linnaeus) Le Jolis

The seaweed species *Ascophyllum nodosum* (Linnaeus) Le Jolis (AN) is harvested from several regions in Ireland, ranging from the far North (Mulroy Bay, Co. Donegal) to the South (Bantry, Cork, Ireland). *A. nodosum* used in animal studies performed at Teagasc, Dublin, Ireland were harvested from the Sligo/Mayo coast in November 2020 and supplied, dried and milled (size of 0.25-1mm) by the Irish company SeaLac Limited (Sligo, Ireland). This seaweed was used for the production of two types of seaweed food additives: AN whole and AN extract.

AN is harvested by boat then transported to a pre-treatment facility for barn drying (Knock, Co. Mayo) and cutting. The fresh AN is placed on racks in a ventilated barn for 24 h and then milled into approximately 0.25–1 mm size pieces. Following milling, a portion of the algae powder is directly supplied to local farms for use in cattle (S1) and sheep (S5) farming. In the AN whole system, a 140-g consumption per cattle per day; and 30 g per sheep per day are assumed. AN whole is fed daily after the nursing period of calves and lamb, until being slaughtered at the age of 2 years (cattle) and 1 year (sheep) for meat production. At these AN inclusion rates, rumen related CH_4_ emissions reductions of 4% have been observed in both cattle and sheep farming^30^.

The remaining harvested AN is processed in a 1:2 AN substrate solution with double distilled H_2_O and transported to a laboratory facility for further fermentation, which lasts for 5 days at a constant temperature of 25 °C. The lactic acid bacteria (LAB) strain used for fermentation is heat deactivated after fermentation at 95 °C for 15 min in a water bath. The resulting fermentate is then transferred to a bioreactor for batch hydrolysis, maintaining a temperature of 25°C for 5 days. After batch hydrolysis, half of the fermented AN undergoes freeze-drying using an FD 80 model (Cuddon Engineering, Marlborough, New Zealand) to produce freeze-dried AN extract. This extract is supplied to cattle farms (S2) and sheep farms (S6) in Ireland. The other half of the fermented AN is dried using a compact chamber dryer (Harter H01 Compact) for 48 h, producing Harter-dried AN extract, which is then supplied to a dairy farm (S3) in Sweden. AN extract was fed daily after the nursing period of calves and lamb, in the amounts of 140 g per cattle and a 60 g per sheep per day. This feeding regime was continued until slaughtered at the age of 2 years (cattle) and 1 year (sheep) for meat production. The freeze-dried AN extract, referred to in Table 1 as AN refined (freeze-dried), can achieve a 9% reduction of enteric CH_4_ emissions in cattle, and 7% in sheep. AN refined (Harter-dried) is fed to dairy cows in Sweden during an animal trial, achieving a reduction of enteric CH_4_ emissions of 9% in dairy cows.

#### *Asparagopsis taxiformis* (Delile) Trevisan 1845

*Asparagopsis taxiformis* (Delile) Trevisan 1845 (AT)’s harvesting was carried out using divers equipped with compressed air tanks, consuming a total of 2390 bar of compressed air to collect 767 kg of fresh biomass. The collected seaweed was loaded onto a 7.2-m fishing boat operating exclusively for this purpose, which consumed 88 L of maritime diesel over two days of harvesting. Once harvested, the fresh biomass was packed in single-use cardboard boxes lined with plastic layers. Each box held approximately 11 kg of seaweed, and a total of 69 boxes, supported by two wooden pallets, were used. The boxes were then transported 400 m from the harbour to freezing chambers using a diesel-powered van, with 523 kg transported on the first day and 725 kg on the second. At the freezing facility, the biomass underwent rapid freezing at − 40°C in a freezing tunnel, followed by storage in -25°C freezing chambers for two to two and a half months. The freezing process utilized approximately 52 kW/h for the tunnel and 32 kW/h for the chambers, ensuring preservation until the next processing stage. No LCA cut-off criteria were applied; all input flows and emissions, regardless of their individual contribution, were included in the analysis.

The frozen biomass was then shipped from Horta to Denmark, passing through Lisbon in a 40-foot refrigerated container. At the freeze-drying facility, the seaweed was processed in cycles lasting 30 to 48 h, with each cycle handling approximately 720 kg of frozen biomass. The process consumed 3940 kW/h of electricity and 580 L of fuel per 24-h period, removing around 90% of the biomass’s water content and producing 20 m^3^ of wastewater daily, treated by public facilities. Finally, the freeze-dried biomass, totalling 75.25 kg, was vacuum sealed in plastic bags and packed into 13 cardboard boxes for delivery, completing the production flow. The algae substitute 0.5% of the dairy cow’s total feed intake and it can reduce enteric CH_4_ production by 59.8%^47^.

#### *Laminaria hyperborea* (Gunnerus) Foslie 1885

The seaweed species *Laminaria hyperborea* (Gunnerus) Foslie 1885 (LH) is harvested in Norway for alginate production, which itself follows a common extraction process described by McHugh^48^. Fresh seaweed undergoes cutting processes to prepare it as raw material for alginate production. Alginate production initiates with acid leaching with HCl, followed by an alkaline extraction where a sodium carbonate solution is blended for 3 h. Cellulose powder is introduced to the mixture after a dewatering process to serve as a filter aid. Subsequently, HCl solution is blended again to obtain precipitated alginic acid, followed by a final dewatering process on a vibrating sieve at cold temperatures. Sodium carbonate is then added to the alginate slurry for neutralization and conversion to sodium alginate. The process generates algae residual, which is pressed, and dried to produce dried LH residual (S7), supplied to sheep farming in Norway. The algae substitute 2.5% of the sheep’s total feed intake and we have assumed a reduction in enteric CH_4_ production by 10%, which will be a best case based on the results by Lind, Hayes^34^.

### Life cycle inventory

The life cycle inventory (LCI) of the three product systems across seven scenarios are presented in Table 2.

**Table 2:** Life cycle inventory.

System		Process	Material	Unit	Scenarios (functional units)						
					S1	S2	S3	S4	S5	S6	S7
					(kg^-1^ beef)	(kg^-1^ beef)	(L^-1^ milk)	(L^-1^ milk)	(kg^-1^ mutton)	(kg^-1^ mutton)	(kg^-1^ mutton)
1	Harvesting										
	Boat	Transport	Diesel	L	0.074	0.074	0.074	0.008	0.008	0.015	0.036
	Truck	Transport	Petrol	L	0.018	0.018	0.004	0.510	0.002	0.004	
		Transport	Diesel	L							0.045
	Diving	Oxygen Refill	Electricity	Wh				0.510			
			Oxygen	kg				0.132			
2	Pretreatment										
	Barn drying	Ventilation	System	p	1.32	1.32	0.263		0.137	0.274	
	Cutting	Cutter	Electricity	Wh	1323	1323	263		137	274	
	Freezing	Freezer	Electricity	Wh				5.766			
3	Refinery										
	Biorefinery	Bioreactor	Electricity	Wh	7297	7297	1451			1511	
			Bacterial Culture	kg		31.8				6.58	
	Alginate Production	Bioreactor	Electricity	Wh							172
		Heating	Heat	MJ							4.15
		Pretreatment	HCL	kg							0.048
		Precipitation	HCL	kg							0.012
		Extraction	NaHCO3	kg							0.051
		Neutralization	NaHCO3	kg							0.005
4	Preservation										
	Freeze drying		Electricity	Wh		9525		559		1973	
			Diesel	L				0.082			
	Harter drying		Electricity	Wh			455				
	Pressing		Electricity	Wh							6850
	LH Drying		Electricity	Wh							6051
5	Packaging										
	Cardboard		Paper	kg	0.886	0.886	0.176	0.004	0.092	0.184	
	Plastic		Polyester	kg	0.042	0.042	0.008	0.000	0.004	0.009	
6	Transport										
	Land Transport		Commercial vehicle	kgkm	30*	30*	30*	30*	30*	30*	30*
	Sea transport		Ferry	kgkm	*	*	*	*	*	*	*
7	Feeding										
	Beef Cattle		Algae feed	kg	1.323	1.323					
	Dairy cow		Algae feed	kg			0.263	0.007			
	Sheep		Algae feed	kg					0.137	0.274	0.137
8	Processing										
	Slaughter		Electricity	Wh	109	109					
			Heat	MJ	0.15	0.15			0.08	0.08	0.08
			Drinking water	kg	2	2			0.5	0.5	0.5
			Truck	kgkm	100	100			100	100	100

*Adjusted in sensitivity analysis.

### Sensitivity analysis

A series of sensitivity analyses were conducted to test the robustness of the results under varying methodological assumptions and key parameters. These analyses focused on three main aspects: the time horizon used to calculate Global Warming Potential (GWP), transport/energy scenarios, and the proportion of indoor housing versus grazing in livestock systems. Given the central role of CH₄ emissions in this study, particular emphasis was placed on comparing short-, mid-, and long-term GWP metrics.

CH₄ is a relatively short-lived GHG with an atmospheric lifetime of roughly 12 years (Myhre et al. 2014). Consequently, its radiative forcing, and thus its weighting relative to CO₂, varies significantly depending on the time horizon chosen for GWP calculations. To capture these differences, we followed the approach advocated by Manzano et al. (2023) and Samsonstuen et al. (2024), reporting results with three GWP time horizons: 20 years (GWP₂₀), 100 years (GWP₁₀₀), and 500 years (GWP₅₀₀). The GWP factors applied were 80.8 (for GWP₂₀), 28 (for GWP₁₀₀), and 7.3 (for GWP₅₀₀), consistent with IPCC (2021) values. GWP₂₀ highlights the near-term warming impact of CH₄, attributing it a higher factor relative to CO₂. GWP₁₀₀ is the standard default approach in most LCA guidelines (including the EF method) and serves as our primary baseline. GWP₅₀₀ illustrates how CH₄’s contribution diminishes substantially over longer timescales, whereas long-lived gases (e.g., CO₂ and N₂O) remain important. By comparing impacts under these three timeframes, we aimed to clarify the degree to which short-lived CH₄ abatement from seaweed feed supplements could deliver near-term benefits versus its importance in the longer-term climate context.

Transport distances and modes were varied to capture realistic supply chains, from local sourcing (under 50 km via road) to interregional routes spanning thousands of kilometres, often involving sea freight. A dedicated sensitivity analysis was conducted to determine how different transport routes and grid mixes might influence the overall life cycle impacts of introducing seaweed feed supplements. Specifically, five distinct routes were modelled to reflect actual production and use cases: (i) a 35 km local road transport, (ii) Ireland-to-Sweden (50 km commercial vehicle + 3150 km ferry + 150 km commercial vehicle), (iii) Ireland-to-France (50 km commercial vehicle + 1020 km ferry + 135 km commercial vehicle), (iv) Norway-to-Sweden (300 km commercial vehicle), and (v) Norway-to-Ireland (2500 km ferry + 50 km commercial vehicle). Since drying and refining seaweed are typically energy-intensive processes, multiple electricity mixes, from fossil-intensive grids to those dominated by renewables, were also tested. By comparing shorter versus longer distances and varying proportions of road versus ferry transport, this analysis aimed to gauge the degree to which localized production or low-carbon energy could offset the emissions associated with algae production and thus alter the overall GWP rankings. Further details on these scenarios and associated assumptions are provided in the Table 2 of the Supplementary material.

The ratio of grazing to indoor housing, with its concomitant reliance on the provision of silage and formulated rations, can likewise alter both the amount of compound feed used and the feasibility of administering seaweed supplements. To capture these effects, a simplified extreme-case sensitivity analysis was performed on the Irish beef system, contrasting 100% grazing (i.e., animals consume fresh grass year-round) against 0% grazing (i.e., animals remain fully indoors and rely exclusively on silage and compound feed). Daily feed consumption was calculated for both fresh grass and indoor feed rations, taking into account a six-month nursing period followed by up to 18 months of fattening prior to slaughter. Although indoor housing presents a controlled environment to deliver novel seaweed additives, greater reliance on grazing can, in some settings where forage quality is high, substantially reduce overall climate impacts by minimizing reliance on processed feeds. By examining the environmental outcomes of these two extremes, the analysis highlights how shifting livestock management toward greater or lesser grazing intensity could affect the net performance of seaweed-based mitigation strategies. Full details on these feeding assumptions and calculations are available in the Supplementary Information. Overall, these sensitivity analyses provide insight into the conditions under which algae-based feeds might meaningfully lower total GHG emissions (especially in short-term horizons) versus scenarios in which the upstream resource demands of seaweed production and processing offset any CH₄ reduction advantage.

## Results

### Overview of impacts

**Fig. 2:**
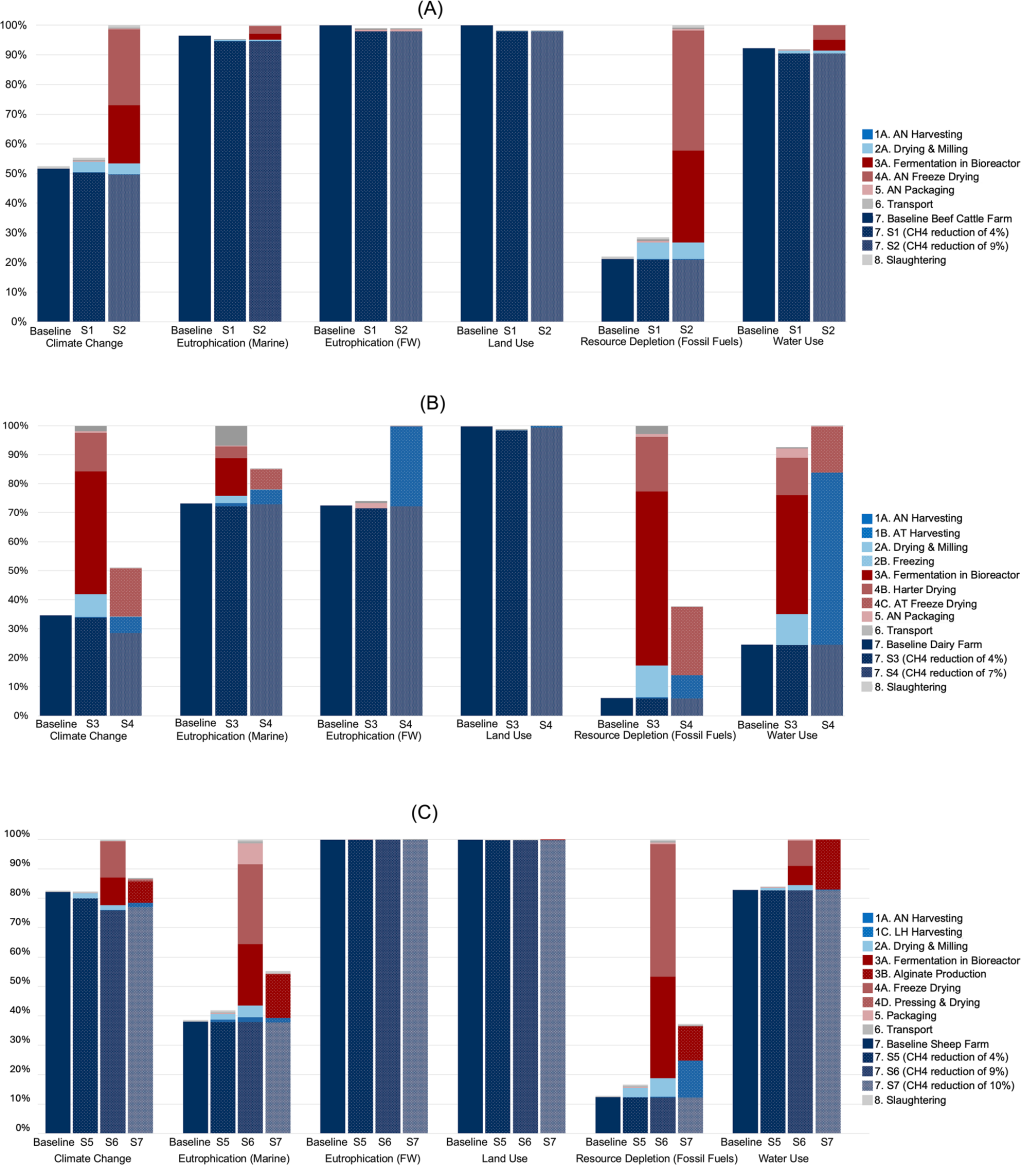
Overview of life cycle impacts of (**A**) the fresh beef production system baseline and scenarios S1 and S2, (**B**) the raw milk production system baseline and scenarios S3 and S4, and (**C**) the fresh mutton production system baseline and scenarios S5, S6 and S7. Impacts are normalised to the worst performing scenario in each comparison and presented across six impact categories of the EF method: climate change, marine and freshwater eutrophication, land use, fossil fuel resource depletion and water use. Supporting impact data are available in Supplementary Tables 3, 4 and 5 in the Supplementary Material.

An overview of results for the beef, milk, and mutton production systems are presented in Fig. 2A, B and C, respectively; supporting impact data are available in Supplementary Tables 3, 4 and 5 in the Supplementary Material. Overall, the baseline scenarios (i.e. production without seaweed supplementation) generally outperform, across most if not all impact categories considered, those including seaweed additives in S1–S7. This outcome arises because the seaweed additives tested in this study reduce enteric CH_4_ emissions by a modest 4–10%, which only affects the climate impact category and has no beneficial effect on the five others. Since the enteric CH_4_ fraction is limited (approximately 37%, 40%, and 60% of total climate impacts in the milk, beef, and mutton baseline systems, respectively), these modest percentage reductions translate into relatively small absolute gains. Moreover, these minor reductions are outweighed by the emissions associated with producing and supplying the seaweed additives in the first place. Consequently, the findings suggest that far greater reductions in enteric CH_4_, achieved by improving seaweed additive effectiveness and/or using lower-impact seaweed products, are needed before meaningful net climate benefits can be realized. However, these potential benefits could be easily offset if the life cycle impacts of seaweed provisioning, remain high or rise.

Across the beef production scenarios, the introduction of seaweed additives exerts a pronounced effect on climate change and resource depletion in S2, where roughly half and 70% of total contributions, respectively, stem from processing the algae, while S1 has minimal (~ 10%) contributions to these two categories. For other impact categories in beef production, the baseline farming system remains the primary source of emissions, largely overshadowing the relatively small contributions from seaweed processing. In the dairy system, S3 dominates multiple categories (climate change, marine eutrophication, resource depletion, and water use) due to energy-intensive processing methods, whereas S4 shows lower proportions in climate and marine eutrophication (under 15–20%) but accounts for around 35% of fossil resource depletion and even higher shares of freshwater eutrophication and water use, driven by the use of compressed air from scuba diving tanks used to harvest the AT. Turning to mutton, S6 incurs substantial burdens, around 20% of climate impacts, approximately 50% of marine eutrophication, 80% of resource depletion, and 20% of water use, whereas S5 and S7 remain low across all these categories. Finally, land use impacts in all systems are dominated by farming activities, with seaweed production contributing negligibly, and a similar trend is observed for freshwater eutrophication except in S4, where the harvesting process elevates this category. Taken together, the pattern is clear: the unsupplemented baseline diets remain the least-burdensome option across every EF-3.1 category, while only the low-tech supplements—AN whole in sheep (S5) and the LH alginate-residue in sheep (S7)—deliver any system-wide impact reductions, and that improvement is modest (≲ 5% in all categories) because the seaweed simply substitutes an equal mass of conventional feed. By contrast, the refined AN scenarios (S2 beef, S3 dairy, S6 mutton) and the freeze-dried AT scenario (S4 dairy) exemplify the trade-off: a ≤ 3% cut in total GWP is achieved at the cost of 25–80% higher fossil-fuel depletion, 20–60% rises in marine and freshwater eutrophication and up to a doubling of water use, all driven by energy-intensive drying, packaging and long-distance transport. Unless those upstream hotspots are radically decarbonised and supply chains shortened, most seaweed additives shift—rather than reduce -environmental burdens through enteric-methane abatement.

Across nearly all baselines and scenarios, except for S2, S3, and S6, the farming life cycle stages dominate overall impacts across most environmental categories. This dominance is particularly pronounced in beef and mutton systems, compared to milk production. The primary reason is that these seaweed additives replace only 2 to 4% of the total feed (depending on the scenario), thus contributing only a small fraction by mass to the overall inputs. In scenarios S2, S3, and S6, however, the novel additives were produced using lab-scale processing and energy intensive preservation techniques. These methods required disproportionately high amounts of material and energy per unit of additive, relative to what would be expected if production were scaled up and optimized. Furthermore, these scenarios are performed in Ireland, where the energy mix is relatively fossil rich, leading to high climate impacts, marine eutrophication potential and fossil resource depletion. As a result, the AN refined scenarios perform worse across all product systems as the GHG emissions resulting from the provision of the additive far outweigh the potential benefits from enteric CH_4_ reduction.

### Climate performance

**Fig. 3:**
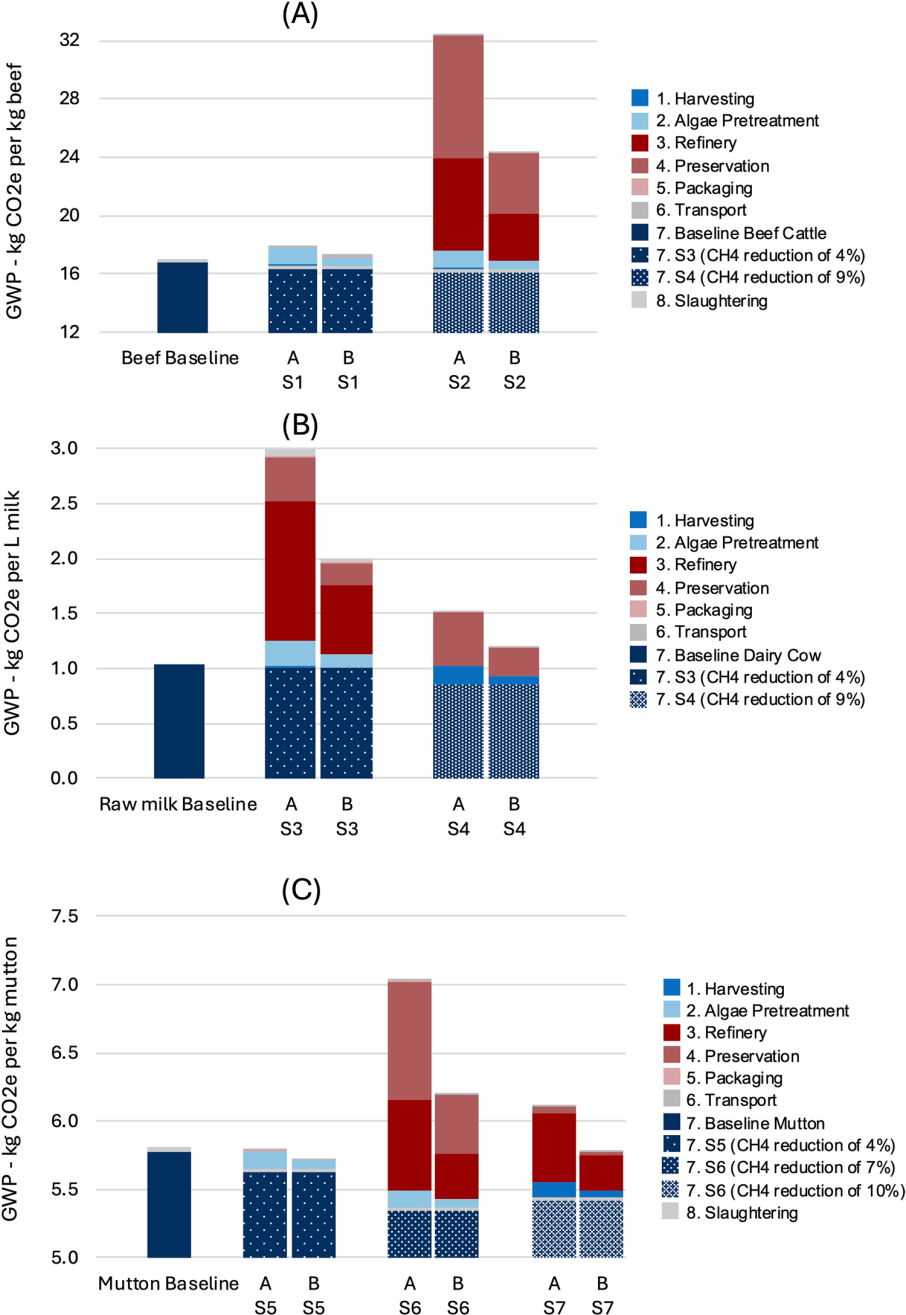
Detailed breakdown of the climate impact of (**A**) the fresh beef production system baseline and scenarios S1 and S2, (**B**) the raw milk production system baseline and scenarios S3 and S4, and (**C**) the fresh mutton production system baseline and scenarios S5, S6 and S7. Two sets of results are presented for each scenario, labelled A and B. A depicts the lab-based processes and unoptimized value chains that were the focus of the present study; B depicts an optimised value chain based on efficiency assumptions reducing impacts by ≈50%. Supporting impact data are available in Supplementary Tables 3, 4 and 5 in the Supplementary Material.

In the beef scenarios, all tested interventions, including the increased efficiency cases presented in column B, consistently performed worse than the baseline. According to the GWP breakdown of the baseline (provided in the Supplementary Information), enteric CH₄ emissions account for approximately 39.5% of total climate impact; thus, a 4–9% reduction in these emissions yields a net climate footprint reduction of ≲3%. Nevertheless, the emissions incurred in sourcing the seaweed additives (S1 and S2), as depicted in Fig. 3A, far exceed the gains achieved through the corresponding reductions in CH₄. The improved efficiency scenarios illustrate that there is considerable potential for further impact mitigation if key parameters, such as transport distance, packaging, processing, and harvesting methods, are optimized. Notably, scenario S2 (AN refined) performs significantly worse than both the baseline and scenario S1 (AN whole), primarily due to the high energy requirements associated with refining and preservation.

In the dairy production system, Fig. 3B, both S3 and S4 yield higher overall impacts compared to the baseline scenario. Although feeding the additives to lactating cows alone achieves a 59% reduction in their individual CH₄ emissions, this benefit translates to only a net 9% decrease for the entire dairy system once calves and heifers are accounted for. Moreover, the high energy demands of processing and drying the AN additive in S3 and freeze-drying the AT additive in S4 further contribute to unfavourable performance relative to the baseline. These findings suggest a broader pattern wherein more energy-intensive additive production methods negatively affect GHG emissions. Accordingly, alternative means of producing AT and AN, such as avoiding freeze-drying, freezing, or energy-intensive drying, warrant exploration to reduce the overall environmental burden of these feed additives. This might not only lower energy use per unit produced but also enhance product stability, potentially leading to greater reductions in CH₄ emissions.

Turning to the mutton production system depicted in Fig. 3C, S6 (AN Refined) exhibits a similarly poor outcome as seen in other algae-refined scenarios (e.g., beef S2 and dairy S3), primarily due to the energy-intensive refinery steps. By contrast, S5 (AN Whole) and S7 (LH Residual) both marginally outperform the baseline, largely because of lower energy consumption during harvesting and preservation, and the absence of refining. Notably, in S7 the additive is a by-product of alginate extraction with comparatively small, allocated impacts, while S5 employs a low impact drying approach instead of freeze-drying. Together, these factors result in net climate benefits, especially in optimized iterations (columns labelled “B”). Finally, relative to beef systems, feeding algae to sheep shows a more favourable GHG profile, likely due to the shorter fattening period for lambs and their lower overall material and energy demands.

### Sensitivity analysis

#### Sensitivity of climate impact method time-horizon

This sensitivity analysis compares the GWP across 20, 100, and 500 year time horizons (GWP₂₀, GWP₁₀₀, and GWP₅₀₀) for both beef and mutton (Table 3). It was not possible to conduct a detailed analysis for dairy cows over the same time horizons because the data^46^ were incompatible with the IPCC (2021) methodology. Nonetheless, similar outcomes would be expected, given dairy cows’ significant CH₄ emissions and longer lifespans, which may yield more pronounced cumulative impacts. Further research using dairy‐specific data would be required to confirm these assumptions.

**Table 3:** Sensitivity analysis on the effect of varying the time horizon of the GWP impact assessment method for the beef product system baseline, S1 and S2, and for the mutton product system baseline, S5, S6 and S7. Impacts are expressed in kgCO_2_e per kg beef or mutton produced. Total = total GHG emissions of each baseline/scenario; Farming CH₄ = CH₄ emissions from both enteric fermentation and manure management; Reduced CH₄ = CH₄ reduction potential of the additives; Farming GHG = total GHG of the farming life cycle stages, excluding CH₄ emissions; Algae Life Cycle GHG = total GHG emissions associated with additive production and transport to farm.

	Beef baseline			S1			S2					
	GWP_20_	GWP_100_	GWP_500_	GWP_20_	GWP_100_	GWP_500_	GWP_20_	GWP_100_	GWP_500_			
Total	30.5	17	9.6	31.1	18.1	11.2	45.8	32.4	25.5			
Farming CH4	19.9	6.74	1.8	18.9	6.29	1.59	18.2	5.96	1.50			
Reduced CH4	0	0	0	0.985	0.451	0.205	1.71	0.785	0.294			
Farming GHG	10.3	10	7.53	10.3	10	7.53	10.3	10	7.53			
Algae Life Cycle GHG	0.295	0.281	0.274	1.85	1.75	2.12	17.3	16.4	16.5			

	Mutton baseline			S5			S6			S7		
	GWP_20_	GWP_100_	GWP_500_	GWP_20_	GWP_100_	GWP_500_	GWP_20_	GWP_100_	GWP_500_	GWP_20_	GWP_100_	GWP_500_
Total	12.6	5.85	2.7	12.3	5.85	2.83	17	10.6	7.64	12.3	6.75	3.23
Farming CH4	9.99	3.39	0.903	9.57	3.23	0.848	9.41	3.17	0.833	8.97	3.02	0.79
Reduced CH4	0	0	0	0.421	0.156	0.055	0.588	0.212	0.07	1.03	0.364	0.113
Farming GHG	2.48	2.39	1.72	2.48	2.39	1.72	2.48	2.39	1.72	2.48	2.39	1.72
Algae Life Cycle GHG	0.079	0.075	0.074	0.24	0.23	0.265	5.43	5.15	5.12	0.872	1.34	0.72

For beef, three scenarios are considered: a baseline (traditional farming), S1 (2% feed substitution with AN whole, and S2 (2% substitution with AN refined. In the baseline scenario, CH₄ dominates, with GWP₂₀ = 19.9, GWP₁₀₀ = 6.74, and GWP₅₀₀ = 1.80. These values illustrate the characteristic ratio for CH₄ across time horizons, whereby GWP₂₀ is roughly three times GWP₁₀₀, which in turn is about three‐to‐four times GWP₅₀₀. Substituting algae‐based feeds lowers CH₄ emissions by 4% in S1 (GWP₂₀ = 18.9, GWP₁₀₀ = 6.29, GWP₅₀₀ = 1.59) and 9% in S2 (GWP₂₀ = 18.2, GWP₁₀₀ = 5.96, GWP₅₀₀ = 1.50). Despite these short‐term gains in CH₄ abatement, the additional GHG emissions from algae production outweigh the CH_4_ reduction benefits, causing the total GWP in both S1 and S2 to exceed the baseline at every time horizon. With GWP₂₀, the high potency of CH₄ makes the reductions appear promising initially, but extending to GWP₁₀₀ or GWP₅₀₀ dilutes CH₄ influence further, revealing the persistent carbon footprint of algae production.

Turning to mutton, the same method is applied to a baseline scenario (traditional farming) and three alternative feed scenarios (S5, S6, S7). S5 and S7 perform better than the baseline at GWP₂₀, but by GWP₁₀₀ and GWP₅₀₀, the baseline either ties (S5) or emerges as superior (S7). None of the alternative feeds surpasses the baseline at GWP₅₀₀. This time‐horizon analysis shows that although S5 and S7 provide modest CH₄ reductions initially, their advantage is offset at longer horizons by algae‐ or residual‐feed‐production emissions. Specifically, S5’s total GHG transitions from slightly below the baseline at GWP₂₀ to matching or exceeding it at longer horizons; S7 achieves the largest CH₄ abatement but also incurs the highest feed‐production‐related GHG. Meanwhile, S6 exceeds the baseline across all time frames. These findings underscore how short‐term metrics (GWP₂₀) amplify the high potency of CH₄, whereas extending to 100‐ or 500‐year horizons diminishes CH₄ impact and highlights the role of non‐CH₄ emissions from feed production.

#### Electricity mix and transport sensitivity

From Table 4, the GWP₁₀₀ varies depending on the electricity mix. Energy mixes with higher renewable energy shares consistently achieve lower GWP values, with Norway’s electricity mix offering the lowest emissions across all scenarios due to its hydropower-dominated grid. Conversely, Ireland’s electricity mix (IE), which relies more heavily on fossil fuels, consistently produces the highest GWP values. Scenarios involving more energy-intensive processes (e.g., S2, S4, S6, and S7) show more pronounced variations across energy mixes, highlighting the significant influence of electricity consumption on GWP outcomes. For example, in S2, transitioning from “IE (Model)” (Ireland) to “NO” (Norway) results in a substantial GWP reduction of 46.7%, while in S4, this reduction is 31.2%. By contrast, scenarios with simplified processes and lower electricity consumption (e.g., S3 and S5) are less sensitive to energy mix changes. For instance, S3 exhibits a 61.2% reduction between “IE (Model)” and “NO,” while S5 shows a smaller reduction of just 1.8%. Interestingly, the impact of energy mix becomes more pronounced in scenarios with higher energy demand, such as S5–S7, where the range of GWP values across energy mixes widens compared to earlier scenarios (S1–S4). This pattern underscores the critical role of renewable energy integration in mitigating GWP, particularly for energy-intensive processes. Furthermore, the consistent relative ranking of electricity mixes across all scenarios highlights the importance of prioritizing low-carbon grids, such as Norway’s, in achieving significant reductions in emissions. These findings emphasize the interplay between energy consumption and grid carbon intensity and demonstrate how decarbonizing electricity grids can substantially improve the environmental performance of energy-intensive systems. For details on modelled electricity mixes, see Supplementary Table 1.

**Table 4:** Sensitivity analysis of using different energy mixes on the Global Warming Potential (GWP_100_, kg CO_2_e) of each scenario, and on different transport scenarios applied to each scenario.

	S1	S2	S3	S4	S5	S6	S7
Electricity							
Electricity mix, IE	18.0	32.3	2.99	1.52	5.79	7.03	6.12
Electricity, low voltage, residual mix, IS	17.7	28.6	2.53	1.40	5.76	6.75	6.1
Electricity, low voltage, Europe without Switzerland	17.3	22.7	1.83	1.22	5.72	6.14	6.09
Electricity, low, Europe	17.3	22.7	1.83	1.22	5.72	6.14	6.09
Norway	16.8	17.2	1.16	1.04	5.68	5.56	6.07
Transport							
Local (35km by vehicle)	18.0	32.3	2.99	1.52	5.79	7.03	6.12
Ireland–Sweden	18.4	32.8	3.04	1.52	5.84	7.08	6.22
Ireland–France	18.3	32.7	3.02	1.52	5.83	7.07	6.21
Norway–Sweden	18.6	33.0	3.08	1.52	5.86	7.10	6.26
Norway–Ireland	19.0	33.4	3.16	1.53	5.9	7.14	6.34

Different transport scenarios for feed additive delivery to farms influence total GWP₁₀₀, though to a much lesser extent than the electricity mix. Locally sourced feed with shorter transport distances consistently exhibits the lowest GWP, as demonstrated by the “Local” route (35 km via commercial vehicle), which has the lowest GWP values across all scenarios (e.g., 18.0 in S1 and 6.12 in S7). In contrast, longer transport distances, such as the “Norway to Ireland” route (approximately 2500 km by ferry and 50 km by commercial vehicle), consistently produce the highest GWP values, reaching 19.0 in S1 and 6.34 in S7. The relative differences between transport scenarios become more pronounced in later scenarios (S5–S7) due to higher energy demands, emphasizing the growing importance of minimizing transport distances as processes become more energy-intensive. Furthermore, vehicle-based transport shows higher emissions per kilometre compared to sea transport, as evidenced by the smaller GWP increase for routes with a significant ferry component, such as the “Ireland to France” route (1020 km by ferry and 185 km by commercial vehicle). This analysis highlights the role of transport distance and mode in determining GWP and underscores that locally produced feed for nearby farms is the most sustainable option, reducing transport-related emissions by up to 5.5% in the depicted scenarios.

#### Grazing sensitivity

This sensitivity analysis evaluates the GWP of livestock systems under two extreme feeding regimes: 100% indoor housing (0% grazing) and 100% grazing, to illustrate the potential impact of varying reliance on grazing. The results reveal a substantial reduction in GWP for livestock systems with 100% grazing compared to those with exclusively indoor housing. For example, in S1, the GWP decreases from 22.1 kg CO₂-eq for 100% indoor housing to 12.2 kg CO₂-eq for 100% grazing, representing a 44.6% reduction. Similarly, in S2, GWP decreases from 21.99 to 12.14 kg CO₂-eq, reflecting a 44.8% reduction. This pattern highlights the lower environmental footprint of high quality grazing systems, which rely less on processed compound feed while maintain similar levels of animal weight gain.

The analysis underscores a key limitation in the implementation of algae-based feed additives. While animal trials in this study were conducted under indoor housing systems to ensure better monitoring and control, integrating algae feed into grazing systems presents practical challenges. During outdoor grazing, livestock primarily consume fresh grass, making it difficult to introduce additives like seaweed. Consequently, it has been suggested that CH₄ mitigation using additives may only be guaranteed during periods of indoor housing, where controlled feeding regimes can be implemented, and much more challenging during pasture grazing though some solutions such as inclusion in salt lick stones have been discussed. Moreover, grazing systems with higher outdoor feeding percentages on high quality pastures inherently have lower GWP due to reduced dependence on environmentally intensive compound feed. However, incorporating algae feed into such systems might necessitate increased compound feed usage, which could offset the benefits of reduced CH₄ emissions. This analysis highlights the importance of accounting for the ratio of indoor to outdoor grazing when evaluating the sustainability of novel feed additives in livestock systems, as this factor significantly influences the potential for GWP reductions. Further research is needed to explore practical strategies for integrating algae feed into grazing systems while maintaining their lower environmental footprint.

## Discussion

### Comparison with peer literature

Although LCAs on algae-based feed supplements for ruminants are still relatively sparse, two recent studies offer helpful points of comparison. The first, by Nilsson and Martin^35^, analysed the production of AT in a land-based cultivation system, concluding that producing 1 kg of dried AT incurred approximately 9.2 kg CO₂e. Under more optimized conditions, incorporating measures such as heat pumps or use of sea salt, the authors estimated that production impacts could potentially drop to between 5.8 and 8.4 kg CO₂e. While these figures are somewhat lower than the impacts observed for our most energy-intensive processes (e.g., freeze-drying in pilot-scale scenarios), the underlying message is similar to our findings: upstream processing can become a critical hotspot, often overshadowing the CH₄ mitigation benefits at the farm level if fossil fuels power the harvesting, drying, or refining steps.

In contrast, Méité, Bayer^36^ extended the scope to include full-system effects of feeding AT to dairy cows in Germany. Their reported reduction in enteric CH₄ emissions, up to 65.5% in high-intake scenarios, ultimately translated into a 23% reduction in overall milk-related GHG emissions. These gains illustrate how a high CH₄ abatement potential (in this case, from AT) can significantly affect the life cycle climate footprint of a ruminant product. Even so, it is noteworthy that the overall GHG reduction was still smaller (i.e., 23%) than the direct CH₄ abatement, underscoring the importance of other GHG hotspots (e.g., feed production, N₂O emissions, and farm energy use) within dairy systems. Interestingly, both Nilsson and Martin^35^ and Méité, Bayer^36^ identified similar upstream hotspots, namely energy use in cultivating and processing the seaweed, that mirror our own findings. However, there are some differences. Nilsson and Martin focus on a land-based AT cultivation method, whereas our study evaluates a range of harvesting and processing routes. Variations in harvest practices naturally shift the relative contributions to the total carbon footprint (e.g., more or less reliance on boats, divers, or specialized equipment). In terms of processing, in both Nilsson and Martin^35^ and our study, energy consumption in drying processes (e.g., freeze-drying, mechanical drying, or other forms of biomass stabilization) emerges as a standout hotspot. In contrast, Méité, Bayer^36^ used secondary data for drying, which was presumably less energy-intensive than our pilot-scale freeze-drying scenarios, and this partially explains why their overall algae production impacts are somewhat lower.

Another key divergence is that both prior LCAs focused on AT, a species known for high CHBr_3_ content and substantial CH₄ abatement (up to 90% in controlled conditions), whereas our study deliberately screened multiple seaweeds, including AN and LH, which are generally less potent in reducing CH₄. As a result, the maximum CH₄ reduction in our scenarios (typically 4–10%) is much lower than what is reported for AT-rich diets (up to 65–90% under certain conditions). These more modest abatement levels translate into smaller overall climate gains once the system-wide emissions are taken into account, making our net results appear less favourable than those reported in the AT-focused studies.

Taken together, these comparisons suggest that seaweed feed supplements can indeed mitigate enteric CH₄ emissions, but both the total abatement potential and the net climate benefit depend critically on the species used and the emissions intensity of upstream processes. While AT can produce higher CH₄ reductions, it may also come with its own set of processing and safety challenges; other seaweeds such as AN or LH might be more readily available and could rely on existing industrial side streams (e.g., alginate residues), but often provide lower CH₄ reductions. In all cases, the degree to which upstream hotspots (e.g., energy-intensive refinement, long-distance transport) can be minimized will be pivotal in determining whether seaweed supplements achieve a net positive environmental outcome.

### System complexity, assumptions, allocations and limitations

The three baseline systems and seven scenarios examined in this study necessarily reflect only one particular configuration of ruminant production practices, illustrating the complexity inherent in real-world systems. In reality, beef, dairy, and sheep farming vary substantially across different regions, even within a single country, due to factors such as climate, regulations, herd genetics, and land availability. For instance, an Irish beef system may prioritize pasture-based feeding with shorter indoor periods than a continental European or Nordic system, while feed mixtures (e.g., silage, maize, concentrates) can differ considerably depending on local availability, seasonal constraints, and farm management objectives. The diversity in livestock support systems, such as the management of heifers and male calves in dairy farms, or varying lambing practices in sheep production, further augments this heterogeneity.

Additionally, to operationalize our life cycle models, a number of assumptions were made regarding transport distances, feeding rates, rearing practices for support animals (e.g., calves, heifers, veal), final weight gain and edible yield rates, and other parameters, often based on expert input or best estimates from project partners. While these choices enabled us to build consistent inventories, they inevitably simplify or aggregate what are often more nuanced on-farm realities. Similarly, allocation challenges arose in accounting for the byproducts of livestock systems and seaweed processing. For instance, we used mass allocation to apportion burdens between meat, offal, and other outputs of slaughter, and likewise allocated a share of alginate production impacts to the algae residue (i.e., LH cake) in Scenario 7. Although these methodological decisions were made with the goal of minimizing biases in impact estimates, the inherent complexity of multi-output systems means such choices could still skew results, especially if different allocation rules (e.g., economic or energy-based) were applied in an alternative modelling framework.

Our analyses relied on a set of LCI datasets widely considered robust and representative within commonly used databases. Nevertheless, any single dataset provides only a snapshot of possible on-farm configurations, and the resulting impact values could shift significantly under alternative management regimes or system definitions. Changes in feeding rates, higher or lower grazing intensities, or distinct approaches to rearing support animals (e.g., veal production, replacement heifers) can markedly alter both absolute and relative GHG burdens. Consequently, while our scenarios offer meaningful insights into the potentials and pitfalls of algae-based feed supplements in mitigating CH₄ emissions, these results should be interpreted with caution. Future work would benefit from more fine-grained, context-specific LCIs that capture the full spectrum of management variations, enabling deeper exploration of how local practices, allocation choices, and system boundaries might enhance, or undermine, the net environmental benefits of seaweed supplements.

A notable limitation of the present study is its relatively narrow system boundary, wherein we primarily investigate the effect of incorporating novel seaweed additives in isolation, though across a number of ruminant product system scenarios. In contrast, Méité, Bayer^36^ evaluated multiple complementary mitigation strategies (e.g., nutritional adjustments, breeding improvements, manure management optimizations) alongside seaweed supplementation. These more holistic models enable an integrated view of how various interventions may act synergistically to yield more substantial reductions in GHG emissions and other environmental burdens. By taking a broader systems perspective, it becomes possible to identify the most promising constellation of on-farm improvements, ranging from diet composition to land-use optimization, that collectively move ruminant production toward lower-impact pathways. Hence, future LCA work should embrace a more comprehensive scope of potential mitigation measures, permitting robust comparisons across multiple strategies and informing decision-makers which combinations of interventions offer the most significant net sustainability gains.

In addition to exploring a broader set of on-farm interventions, there remains the challenge of fully characterizing complex emission flows. For instance, although CHBr_3_ is a key active compound linked to the enteric CH₄-inhibiting properties of certain seaweeds, its atmospheric fate and potential contribution to ozone depletion or subsequent GHG formation are not yet well-understood. Because these emissions lie outside conventional inventory frameworks and are subject to major data gaps, they were excluded from the present study, in line with most contemporary LCAs of seaweed-based solutions. Moreover, the low technological readiness level (TRL) of many seaweed harvesting and processing methods underscores the nascent nature of this domain. Unlike established feed products that operate at industrial scales and benefit from decades of process optimization, seaweed-based supplements in this study were largely produced under pilot or laboratory conditions. As a result, their current environmental footprints may not reflect the improvements possible under larger-scale, technologically mature operations. Until these production systems are scaled and optimized to match existing feed supply chains, direct comparisons and extrapolations from lab-scale processes to global livestock sectors will necessarily entail considerable uncertainty.

### Broader constraints on deployment and scale-up

Recent estimates place the global cattle population at around 942.6 million^49^ and the European Union’s at 74 million^50^. Based on the present study’s modelled feed intake rate (approximately 140 g dried or 1 kg fresh weight per cow per day), meeting the EU’s annual requirements alone would demand around 25 million tons fresh weight, and the global figure could be ten times higher. Yet, in 2021, total seaweed production was estimated only at 36.3 million tons^51^ largely from aquaculture of kelp in Asia, with *Asparagopsis* spp. contributing a fraction of a percent. This illustrates the significant challenges in scaling up *Asparagopsis* spp. production to reduce enteric methane on a meaningful level, not only mandatorily demanding the use of offshore aquaculture for maximum yield as soon as that technology is unveiled, but also the adoption of alternative processing methods that retain higher CHBr_3_ levels with a lower GHG footprint, enabling higher anti-methanogenic effects with less *Asparagopsis* spp. biomass.

A further complication is the alien (invasive) status of *Asparagopsis* spp., which triggers stringent EU regulations aimed at preventing and controlling the spread of non-native species (e.g., Regulation 1143/2014). Seaweed aquaculture remains nascent in Europe, and legislation governing alien species in aquaculture (e.g., Regulation 708/2007) is still evolving. Consequently, even if large-scale cultivation could be achieved, ensuring regulatory compliance and minimizing ecological impacts would present substantial hurdles for *Asparagopsis*-based CH₄ mitigation efforts.

## Conclusions

In this study, we explored the potential for methane mitigation through the use of diverse seaweed species and pretreatment methods applied across multiple ruminant production systems. The results of this study underscore the challenging trade-offs inherent in using seaweed-based feed supplements for reducing enteric CH₄ emissions. While certain algae additives can effectively mitigate CH₄ production, particularly over short time horizons (e.g., GWP₂₀), the broader life cycle impacts often offset these gains. In several scenarios, especially those involving energy-intensive processing or lengthy transport, the net GHG footprint rose relative to a conventional baseline. Even where moderate CH₄ emission reductions were observed, these abatement benefits translated to only marginal changes in total climate impacts once other GHG contributors (e.g., CO₂, N₂O) were included.

Nevertheless, the study also reveals that some algae supplementation scenarios, particularly those involving low impact harvesting or by-product use (e.g., LH residue from alginate production), can modestly outperform conventional production. The improved performance in these cases is primarily attributable to minimal processing requirements and lower allocated burdens. Taken together, these findings highlight that algae-based feeds must be developed and integrated under optimized, low-emission conditions if they are to contribute meaningfully to climate change mitigation.

Looking ahead, further research is needed to refine the life cycle data, expand system boundaries, and investigate complementary management improvements, such as optimized feed rations, precision grazing, and manure management, to maximize synergies in livestock systems. Additionally, future studies should explore the long-term implications and scalability of seaweed-based solutions, including the fate of CHBr_3_ emissions and the technological maturity of algae production. Ultimately, while algae feed supplements can form part of a broader strategy to decarbonize ruminant systems, their real-world viability will hinge on achieving lower upstream impacts and aligning with complementary on-farm mitigation measures.

## Supplementary Information

Supplementary Information.

## Data Availability

The datasets used and/or analysed during the current study available from the corresponding author on reasonable request.
