# Supplementary material for: Seaweed as a climate fix for meat and dairy production: an LCA perspective
Source: Sci Rep. 2025 Sep 18;15:32597. doi: 10.1038/s41598-025-18322-1 (PMC12446431; doi:10.1038/s41598-025-18322-1)
Supplement: Supplementary file 1 — Supplementary Information. [file 41598_2025_18322_MOESM1_ESM.docx]

**Supplementary Material:**

**Seaweed as a Climate Fix for Meat and Dairy Production: An LCA Perspective**

**Baseline scenarios**


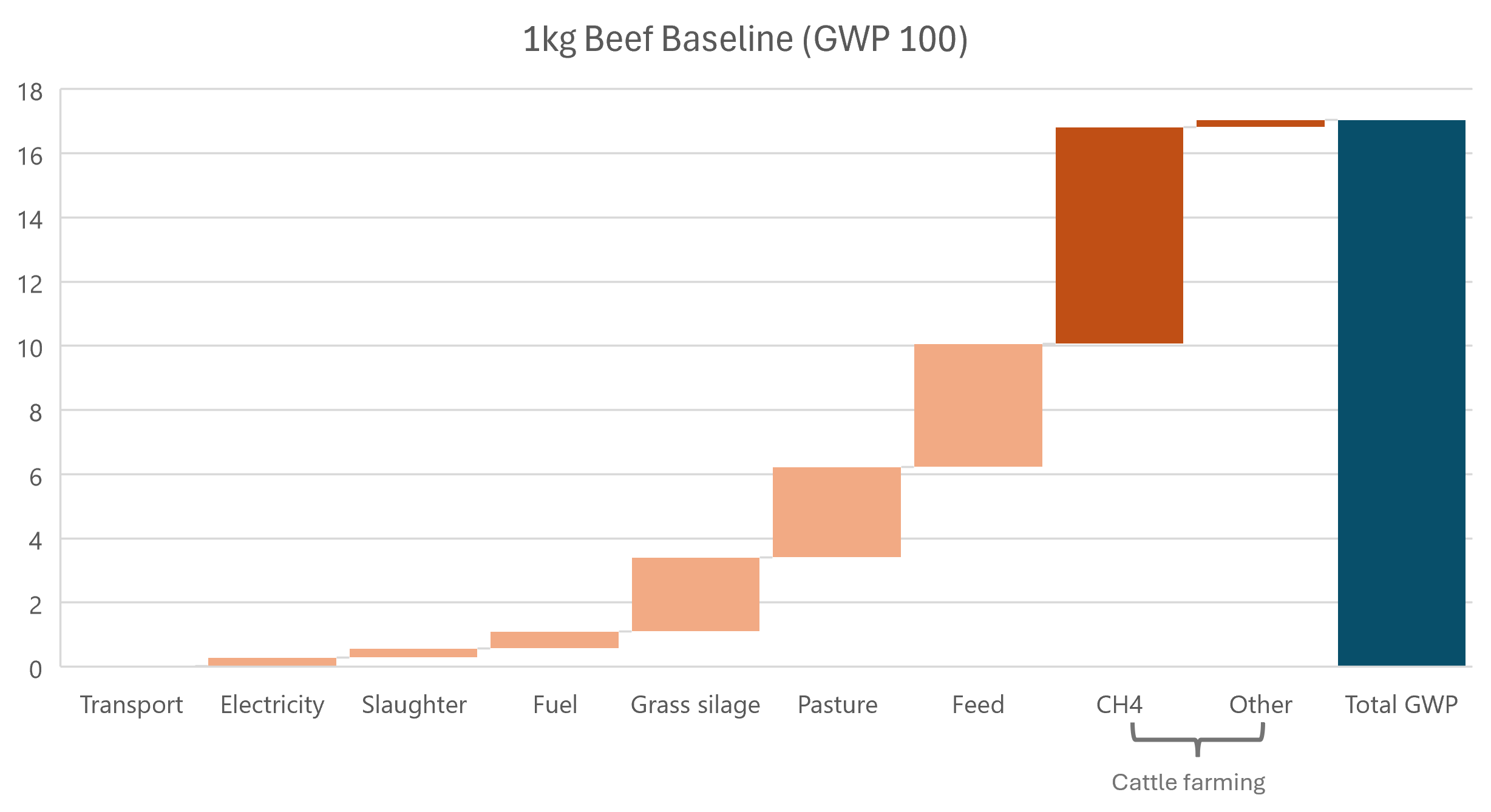


**Supplementary Figure 1:** Breakdown of life cycle climate impacts of the cattle farming process (1 kg Beef meat, fresh, from beef cattle, at slaughterhouse/IE Mass (of project Agri-footprint - mass allocation)


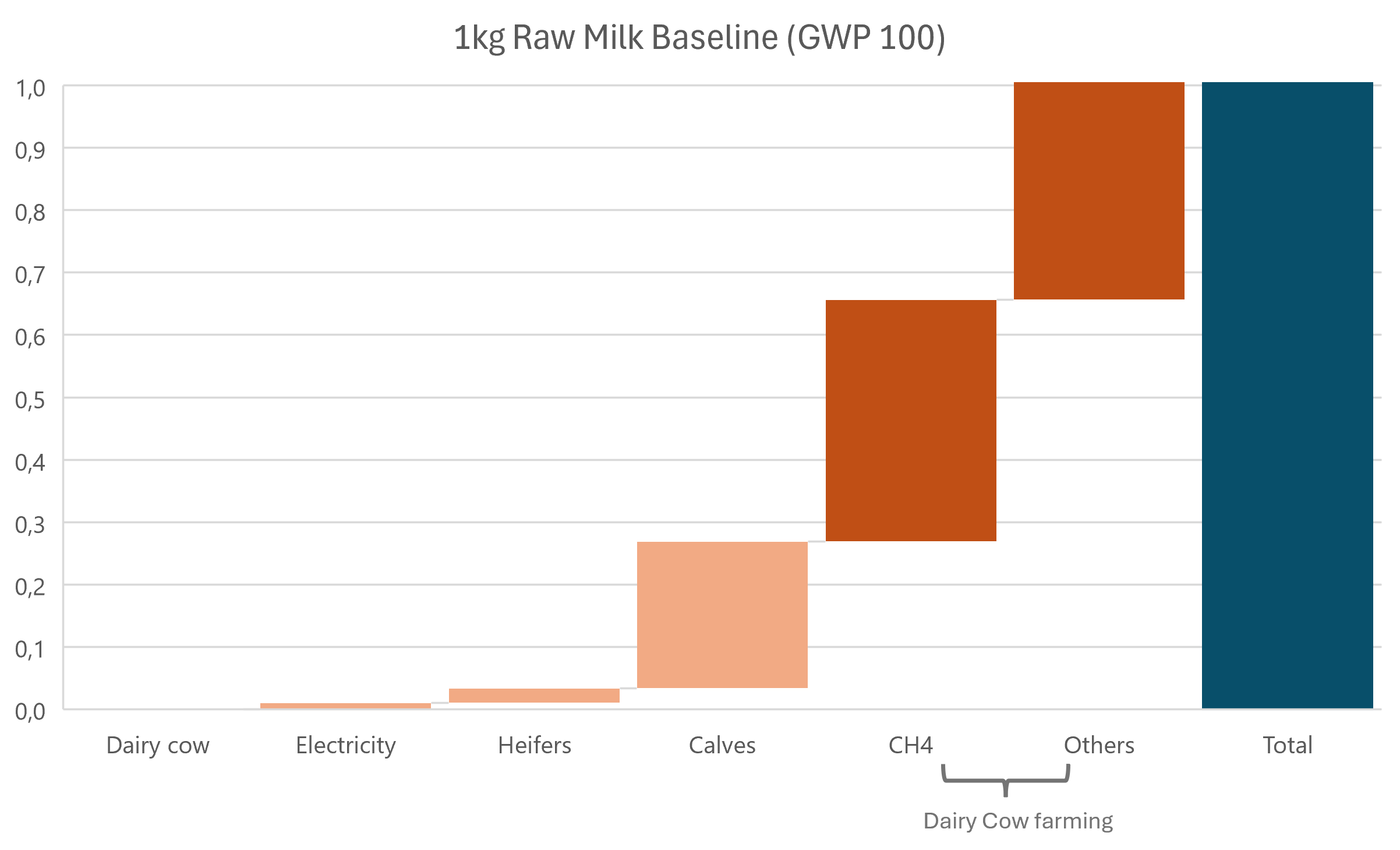


**Supplementary Figure 2**: Breakdown of life cycle climate impacts of the dairy cow farming (1 kg Milk raw, at farm {DK} Economic, U (of project Agri-footprint - economic - unit). Note: 1kg of raw milk is assumed to be equivalent to 1L in the study.


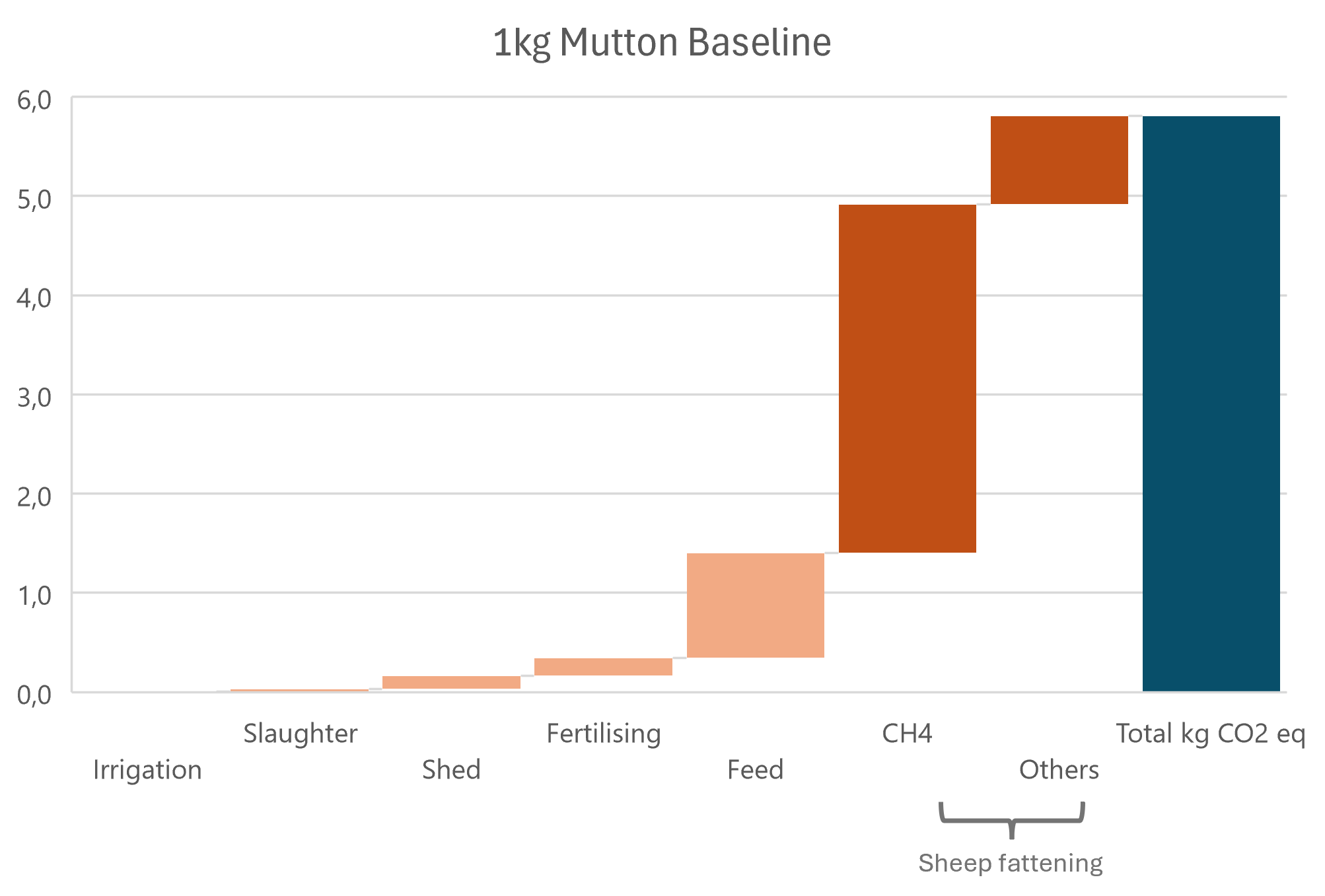


**Supplementary Figure 3**: Breakdown of life cycle climate impacts of the sheep farming process (1 kg Sheep for slaughtering, live weight {RoW}| sheep production, for meat | APOS, U (of project Ecoinvent 3 - allocation at point of substitution - unit))

**Supplementary Table 1.** Electricity sensitivity: data source and selection

| Electricity Scenario | | Data Entry | | Source | |
| --- | --- | --- | --- | --- | --- |
| Electricity | | Electricity mix, AC, consumption mix, at consumer, < 1kV IE S System - Copied from ELCD | | Agrifootprint | |
| Electricity mix, IE | | electricity, low voltage, residual mix \| electricity, low voltage \| APOS, U - IS | | Ecoinvent | |
| Electricity, low voltage, residual mix, IE | | market group for electricity, low voltage \| electricity, low voltage \| APOS, U - Europe without Switzerland | | Ecoinvent | |
| Electricity, low voltage, Europe without Switzerland | | market group for electricity, low voltage \| electricity, low voltage \| APOS, U - RER | | Ecoinvent | |
| Electricity, low, Europe | | Electricity mix, AC, consumption mix, at consumer, < 1kV NO S System - Copied from ELCD | | Agrifootprint | |
|  |  | |  | |  |

**Supplementary Table 2.** Transport Sensitivity: transport routes and distance

| Route | From |  | To |  | Type | Distance | Unit |
| --- | --- | --- | --- | --- | --- | --- | --- |
| Ireland Local | Ireland | Manufacturer | Ireland | Farm | Commercial Vehicle | 50 | km |
| Ireland to Sweden | Ireland | Manufacturer | Ireland | Dublin Port | Commercial Vehicle | 50 | km |
|  | Ireland | Dublin Port | Sweden | Gothenburg Port | Ferry | 3150 | km |
|  | Sweden | Gothenburg Port | Sweden | Farm in Halland County (West Coast) | Commercial Vehicle | 150 | km |
| Ireland to France | Ireland | Manufacturer | Ireland | Farm | Commercial Vehicle | 50 | km |
|  | Ireland | Dublin Port | France | Cherbourg port | Ferry | 1020 | km |
|  | France | Cherbourg port | France | Normandy Farming Region (e.g., Caen, Le Havre) | Commercial Vehicle | 135 | km |
| Norway to Sweden | Norway | Oslo | Sweden | Farm in Halland County (West Coast) | Commercial Vehicle | 300 | km |
| Norway to Ireland | Norway | Manufacturer | Norway | Bergen Port | Commercial Vehicle | 400 | km |
|  | Norway | Bergen Port | Ireland | Dublin Port | Ferry | 2500 | km |
|  | Ireland | Dublin Port | Ireland | Leinster farm | Commercial Vehicle | 50 | km |

| **Supplementary Table 3.**  Life cycle impacts of the production of 1kg bone-free beef in the baseline system and two scenarios, S1 and S2, showing both impact data and relative contributions (**%**) of each life cycle stage normalised to the worst performing production system. | | | | | | | | | | | | | | | | | | | | |
| --- | --- | --- | --- | --- | --- | --- | --- | --- | --- | --- | --- | --- | --- | --- | --- | --- | --- | --- | --- | --- |
|  | **Climate change (kg CO2 eq)** | | | | | |  | **Eutrophication, marine (kg N eq)** | | | | | |  | **Eutrophication, freshwater (kg P eq)** | | | | | |
| **Life Cycle Stage** | **Baseline** |  | **S1** |  | **S2** |  |  | **Baseline** |  | **S1** |  | **S2** |  |  | **Baseline** |  | **S1** |  | **S2** |  |
| 1A. AN Harvesting |  |  | 4.31E-02 | **0.1%** | 4.31E-02 | **0.1%** |  |  |  | 3.69E-04 | **0.2%** | 3.69E-04 | **0.2%** |  |  |  | 4.33E-07 | **0.0%** | 4.33E-07 | **0.0%** |
| 2A. Drying & Milling |  |  | 1.16E+00 | **3.6%** | 1.16E+00 | **3.6%** |  |  |  | 8.63E-04 | **0.4%** | 8.63E-04 | **0.4%** |  |  |  | 2.68E-07 | **0.0%** | 2.68E-07 | **0.0%** |
| 3. Fermentation in Bioreactor |  |  |  |  | 6.38E+00 | **19.7%** |  |  |  |  |  | 4.50E-03 | **2.0%** |  |  |  |  |  | 1.82E-07 | **0.0%** |
| 4A. AN Freeze Drying |  |  |  |  | 8.33E+00 | **25.8%** |  |  |  |  |  | 5.88E-03 | **2.7%** |  |  |  |  |  | 2.38E-07 | **0.0%** |
| 5. AN Packaging |  |  | 7.81E-02 | **0.2%** | 7.81E-02 | **0.2%** |  |  |  | 8.25E-05 | **0.0%** | 8.25E-05 | **0.0%** |  |  |  | 1.88E-05 | **0.5%** | 1.88E-05 | **0.5%** |
| 6. Transport |  |  | 8.54E-02 | **0.3%** | 8.54E-02 | **0.3%** |  |  |  | 1.47E-04 | **0.1%** | 1.47E-04 | **0.1%** |  |  |  | 1.29E-05 | **0.4%** | 1.29E-05 | **0.4%** |
| 7. Baseline Beef Cattle Farm | 1.68E+01 | **51.8%** |  |  |  |  |  | 2.14E-01 | **96.4%** |  |  |  |  |  | 3.44E-03 | **100.0%** |  |  |  |  |
| 7. S1 (CH4 reduction of 4%) |  |  | 1.63E+01 | **50.4%** |  |  |  |  |  | 2.09E-01 | **94.5%** |  |  |  |  |  | 3.37E-03 | **98.0%** |  |  |
| 7. S2 (CH4 reduction of 9%) |  |  |  |  | 1.60E+01 | **49.4%** |  |  |  |  |  | 2.09E-01 | **94.5%** |  |  |  |  |  | 3.37E-03 | **98.0%** |
| 8. Slaughtering | 2.79E-01 | **0.9%** | 2.79E-01 | **0.9%** | 2.79E-01 | **0.9%** |  | 2.57E-04 | **0.1%** | 2.57E-04 | **0.1%** | 2.57E-04 | **0.1%** |  | 2.10E-08 | **0.0%** | 2.10E-08 | **0.0%** | 2.10E-08 | **0.0%** |
| **Total** | **1.70E+01** | **52.7%** | **1.80E+01** | **55.5%** | **3.23E+01** | **100.0%** |  | **2.14E-01** | **96.5%** | **2.11E-01** | **95.3%** | **2.21E-01** | **100.0%** |  | **3.44E-03** | **100.0%** | **3.40E-03** | **98.9%** | **3.40E-03** | **99.0%** |

|  | **Land use (Pt)** | | | | | |  | **Resource use, fossils (MJ)** | | | | | |  | **Water use (m3 depriv.)** | | | | | |
| --- | --- | --- | --- | --- | --- | --- | --- | --- | --- | --- | --- | --- | --- | --- | --- | --- | --- | --- | --- | --- |
| **Life Cycle Stage** | **Baseline** |  | **S1** |  | **S2** |  |  | **Baseline** |  | **S1** |  | **S2** |  |  | **Baseline** |  | **S1** |  | **S2** |  |
| 1A. AN Harvesting |  |  | 7.27E-02 | **0.0%** | 7.27E-02 | **0.0%** |  |  |  | 5.94E-01 | **0.2%** | 5.94E-01 | **0.2%** |  |  |  | 2.80E-04 | **0.0%** | 2.80E-04 | **0.0%** |
| 2A. Drying & Milling |  |  | 4.15E-01 | **0.0%** | 4.15E-01 | **0.0%** |  |  |  | 1.43E+01 | **5.7%** | 1.43E+01 | **5.7%** |  |  |  | 1.25E-01 | **1.0%** | 1.25E-01 | **1.0%** |
| 3. Fermentation in Bioreactor |  |  |  |  | 0.00E+00 | **0.0%** |  |  |  |  |  | 7.81E+01 | **31.0%** |  |  |  |  |  | 4.81E-01 | **3.7%** |
| 4A. AN Freeze Drying |  |  |  |  | 0.00E+00 | **0.0%** |  |  |  |  |  | 1.02E+02 | **40.5%** |  |  |  |  |  | 6.28E-01 | **4.9%** |
| 5. AN Packaging |  |  | 1.97E-01 | **0.0%** | 1.97E-01 | **0.0%** |  |  |  | 1.26E+00 | **0.5%** | 1.26E+00 | **0.5%** |  |  |  | 3.77E-02 | **0.3%** | 3.77E-02 | **0.3%** |
| 6. Transport |  |  | 6.14E-01 | **0.1%** | 6.14E-01 | **0.1%** |  |  |  | 1.29E+00 | **0.5%** | 1.29E+00 | **0.5%** |  |  |  | 6.80E-03 | **0.1%** | 6.80E-03 | **0.1%** |
| 7. Baseline Beef Cattle Farm | 9.92E+02 | **100.0%** |  |  |  |  |  | 5.34E+01 | **21.2%** |  |  |  |  |  | 1.19E+01 | **92.3%** |  |  |  |  |
| 7. S1 (CH4 reduction of 4%) |  |  | 9.72E+02 | **98.0%** |  |  |  |  |  | 5.26E+01 | **20.9%** |  |  |  |  |  | 1.17E+01 | **90.5%** |  |  |
| 7. S2 (CH4 reduction of 9%) |  |  |  |  | 9.72E+02 | **98.0%** |  |  |  |  |  | 5.26E+01 | **20.9%** |  |  |  |  |  | 1.17E+01 | **90.5%** |
| 8. Slaughtering | 0.00E+00 | **0.0%** | 0.00E+00 | **0.0%** | 0.00E+00 | **0.0%** |  | 1.92E+00 | **0.8%** | 1.92E+00 | **0.8%** | 1.92E+00 | **0.8%** |  | -4.99E-02 | **-0.4%** | -4.99E-02 | **-0.4%** | -4.99E-02 | **-0.4%** |
| **Total** | **9.92E+02** | **100.0%** | **9.74E+02** | **98.1%** | **9.74E+02** | **98.1%** |  | **5.53E+01** | **22.0%** | **7.19E+01** | **28.5%** | **2.52E+02** | **100.0%** |  | **1.19E+01** | **91.9%** | **1.18E+01** | **91.4%** | **1.29E+01** | **100.0%** |

| **Supplementary Table 4.**  Life cycle impacts of the production of 1L raw milk in the baseline system and two scenarios, S3 and S4, showing both impact data and relative contributions (**%**) of each life cycle stage normalised to the worst performing production system. | | | | | | | | | | | | | | | | | | |
| --- | --- | --- | --- | --- | --- | --- | --- | --- | --- | --- | --- | --- | --- | --- | --- | --- | --- | --- |
|  | **Climate change (kg CO2 eq)** | | | | | | **Eutrophication, marine (kg N eq)** | | | | | | **Eutrophication, freshwater (kg P eq)** | | | | | |
| **Life Cycle Stage** | **Baseline** |  | **S3** |  | **S4** |  | **Baseline** |  | **S3** |  | **S4** |  | **Baseline** |  | **S3** |  | **S4** |  |
| 1A. AN Harvesting |  |  | 8.57E-03 | **0.3%** |  |  |  |  | 7.34E-05 | **1.1%** |  |  |  |  | 8.60E-08 |  |  |  |
| 1B. AT Harvesting |  |  |  |  | 1.66E-01 | **5.5%** |  |  |  |  | 3.46E-04 | **5.0%** |  |  |  |  | 6.01E-05 | **27.6%** |
| 2A. Drying & Milling |  |  | 2.31E-01 | **7.7%** |  |  |  |  | 1.72E-04 | **2.5%** |  |  |  |  | 5.33E-08 | **0.0%** |  |  |
| 2B. Freezing |  |  |  |  | 3.61E-03 | **0.1%** |  |  |  |  | 2.55E-06 | **0.0%** |  |  |  |  | 1.03E-10 | **0.0%** |
| 3. Fermentation in Bioreactor |  |  | 1.27E+00 | **42.4%** |  |  |  |  | 8.95E-04 | **13.0%** |  |  |  |  | 3.62E-08 | **0.0%** |  |  |
| 4A. AN Freeze Drying |  |  | 3.98E-01 | **13.3%** |  |  |  |  | 2.81E-04 | **4.1%** |  |  |  |  | 1.14E-08 | **0.0%** |  |  |
| 4C. AT Freeze Drying |  |  |  |  | 4.96E-01 | **16.6%** |  |  |  |  | 4.06E-04 | **7.0%** |  |  |  |  | 8.47E-08 | **0.0%** |
| *5. AN Packaging* |  |  | 1.55E-02 | **0.5%** |  |  |  |  | 1.64E-05 | **0.2%** |  |  |  |  | 3.74E-06 | **1.7%** |  |  |
| 5. Packaging |  |  |  | **0.5%** | 1.31E-03 | **0.0%** |  |  |  | **0.2%** | 1.38E-06 | **0.0%** |  |  |  | **1.7%** | 3.15E-07 | **0.1%** |
| 6. Transport |  |  | 5.73E-02 | **1.9%** | 4.24E-04 | **0.0%** |  |  | 4.71E-04 | **6.9%** | 7.32E-07 | **0.0%** |  |  | 1.67E-06 | **0.8%** | 6.39E-08 | **0.0%** |
| 7. Baseline Dairy Farm | 1.04E+00 | **34.7%** |  |  |  |  | 5.03E-03 | **73.2%** |  |  |  |  | 1.58E-04 | **72.5%** |  |  |  |  |
| 7. S3 (CH_4_ reduction of 4%) |  |  | 1.01E+00 | **33.8%** |  |  |  |  | 4.96E-03 | **72.2%** |  |  |  |  | 1.56E-04 | **71.5%** |  |  |
| 7. S4 (CH_4_ reduction of 9%) |  |  |  |  | 8.53E-01 | **28.5%** |  |  |  |  | 5.01E-03 | **72.9%** |  |  |  |  | 1.57E-04 | **72.2%** |
| **Total** | **1.04E+00** | **34.7%** | **2.99E+00** | **100.0%** | **1.52E+00** | **50.9%** | **5.03E-03** | **73.2%** | **6.87E-03** | **100.0%** | **5.77E-03** | **83.9%** | **1.58E-04** | **72.5%** | **1.61E-04** | **74.1%** | **2.18E-04** | **100.0%** |
|  | **Land use (Pt)** | | | | | | **Resource use, fossils (MJ)** | | | | | | **Water use (m3 depriv.)** | | | | | |
| **Life Cycle Stage** | **Baseline** |  | **S3** |  | **S4** |  | **Baseline** |  | **S3** |  | **S4** |  | **Baseline** |  | **S3** |  | **S4** |  |
| 1A. AN Harvesting |  |  | 1.44E-02 | **0.0%** |  |  |  |  | 1.18E-01 | **0.5%** |  |  |  |  | 5.56E-05 | **0.0%** |  |  |
| 1B. AT Harvesting |  |  |  |  | 3.32E-01 | **0.6%** |  |  |  |  | 2.07E+00 | **8.0%** |  |  |  |  | 1.38E-01 | **59.3%** |
| 2A. Drying & Milling |  |  | 8.26E-02 | **0.2%** |  |  |  |  | 2.83E+00 | **11.0%** |  |  |  |  | 2.48E-02 | **10.7%** |  |  |
| 2B. Freezing |  |  |  |  | 0.00E+00 | **0.0%** |  |  |  |  | 1.91E-03 | **0.0%** |  |  |  |  | 1.18E-05 | **0.0%** |
| 3. Fermentation in Bioreactor |  |  | 0.00E+00 | **0.0%** |  |  |  |  | 1.55E+01 | **60.0%** |  |  |  |  | 9.57E-02 | **41.1%** |  |  |
| 4A. AN Freeze Drying |  |  | 0.00E+00 | **0.0%** |  |  |  |  | 4.87E+00 | **18.8%** |  |  |  |  | 3.00E-02 | **12.9%** |  |  |
| 4C. AT Freeze Drying |  |  |  |  | 3.87E-12 | **0.0%** |  |  |  |  | 6.08E+00 | **23.5%** |  |  |  |  | 3.69E-02 | **15.9%** |
| *5. AN Packaging* |  |  | 3.91E-02 | **0.1%** |  |  |  |  | 2.50E-01 | **1.0%** |  |  |  |  | 7.49E-03 | **3.2%** |  |  |
| 5. Packaging |  |  |  | **0.1%** | 3.33E-03 | **0.0%** |  |  |  | **1.0%** | 2.12E-02 | **0.1%** |  |  |  | **3.2%** | 6.36E-04 | **0.3%** |
| 6. Transport |  |  | 9.63E-02 | **0.2%** | 3.05E-03 | **0.0%** |  |  | 7.34E-01 | **2.8%** | 6.39E-03 | **0.0%** |  |  | 1.14E-03 | **0.5%** | 3.38E-05 | **0.0%** |
| 7. Baseline Dairy Farm | 5.36E+01 | **99.7%** |  |  |  |  | 1.56E+00 | **6.0%** |  |  |  |  | 5.74E-02 | **24.6%** |  |  |  |  |
| 7. S3 (CH_4_ reduction of 4%) |  |  | 5.29E+01 | **98.4%** |  |  |  |  | 1.54E+00 | **6.0%** |  |  |  |  | 5.67E-02 | **24.3%** |  |  |
| 7. S4 (CH_4_ reduction of 9%) |  |  |  |  | 5.34E+01 | **99.4%** |  |  |  |  | 1.56E+00 | **6.0%** |  |  |  |  | 5.72E-02 | **24.5%** |
| **Total** | **5.36E+01** | **99.7%** | **5.31E+01** | **98.8%** | **5.38E+01** | **100.0%** | **1.56E+00** | **6.0%** | **2.59E+01** | **100.0%** | **9.74E+00** | **37.7%** | **5.74E-02** | **24.6%** | **2.16E-01** | **92.7%** | **2.33E-01** | **100.0%** |

| **Supplementary Table 5.**  Life cycle impacts of the production of 1kg of boneless mutton in the baseline system and three scenarios, S5, S6 and S7, showing both impact data and relative contributions (**%**) of each life cycle stage normalised to the worst performing production system. | | | | | | | | | | | | | | | | | | | | | | | | |
| --- | --- | --- | --- | --- | --- | --- | --- | --- | --- | --- | --- | --- | --- | --- | --- | --- | --- | --- | --- | --- | --- | --- | --- | --- |
|  | **Climate change (kg CO2 eq)** | | | | | | | | **Eutrophication, marine (kg N eq)** | | | | | | | | **Eutrophication, freshwater (kg P eq)** | | | | | | | |
| **Life Cycle Stage** | **Baseline** |  | **S5** |  | **S6** |  | **S7** |  | **Baseline** |  | **S5** |  | **S6** |  | **S7** |  | **Baseline** |  | **S5** |  | **S6** |  | **S7** |  |
| 1A. AN Harvesting |  |  | 4.47E-03 | **0.1%** | 4.47E-03 | **0.1%** |  |  |  |  | 3.82E-05 | **0.9%** | 7.65E-05 | **1.7%** |  |  |  |  | 4.48E-08 | **0.0%** | 8.96E-08 | **0.0%** |  |  |
| 1B. LH Harvesting |  |  |  |  |  |  | 1.04E-01 | **1.5%** |  |  |  |  |  |  | 6.52E-05 | **1.5%** |  |  |  |  |  |  | 5.98E-06 | **0.0%** |
| 2A. Drying & Milling |  |  | 1.21E-01 | **1.7%** | 1.21E-01 | **1.7%** |  |  |  |  | 8.94E-05 | **2.0%** | 1.79E-04 | **4.0%** |  |  |  |  | 2.78E-08 | **0.0%** | 1.04E-04 | **0.0%** |  |  |
| 3A. Fermentation in Bioreactor |  |  |  |  | 6.61E-01 | **9.4%** |  |  |  |  |  |  | 9.33E-04 | **20.9%** |  |  |  |  |  |  | 3.77E-08 | **0.0%** |  |  |
| 3B. Alginate Production |  |  |  |  |  |  | 5.12E-01 | **7.3%** |  |  |  |  |  |  | 6.66E-04 | **14.9%** |  |  |  |  |  |  | 2.25E-04 | **0.0%** |
| 4A. Harter Drying |  |  |  |  | 8.63E-01 | **12.3%** |  |  |  |  |  |  | 1.22E-03 | **27.2%** |  |  |  |  |  |  | 4.93E-08 | **0.0%** |  |  |
| 4B. Pressing & Drying |  |  |  |  |  |  | 4.37E-02 | **0.6%** |  |  |  |  |  |  | 4.23E-06 | **0.1%** |  |  |  |  |  |  | 1.71E-10 | **0.0%** |
| 5. Packaging |  |  | 8.08E-03 | **0.1%** | 8.08E-03 | **0.1%** | 8.08E-03 | **0.1%** |  |  | 8.54E-06 | **0.2%** | 3.13E-04 | **7.0%** | 8.54E-06 | **0.2%** |  |  | 3.63E-03 | **0.5%** | 3.89E-06 | **0.0%** | 1.95E-06 | **0.0%** |
| 6. Transport |  |  | 8.84E-03 | **0.1%** | 8.84E-03 | **0.1%** |  |  |  |  | 1.52E-05 | **0.3%** | 3.05E-05 | **0.7%** |  |  |  |  | 1.33E-06 | **0.0%** | 2.66E-06 | **0.0%** |  |  |
| 6B. Shipping to Norwegian farm |  |  |  |  |  |  | 4.85E-03 | **0.1%** |  |  |  |  |  |  | 3.75E-06 | **0.1%** |  |  |  |  |  |  | 2.24E-07 | **0.0%** |
| 7. Baseline Sheep Farm | 5.78E+00 | **82.2%** |  |  |  |  |  |  | 1.70E-03 | **38.0%** |  |  |  |  |  |  | 7.11E-01 | **99.6%** |  |  |  |  |  |  |
| 7. S5 (CH4 reduction of 4%) |  |  | 5.62E+00 | **79.9%** |  |  |  |  |  |  | 1.69E-03 | **37.8%** |  |  |  |  |  |  | 7.10E-01 | **99.9%** |  |  |  |  |
| 7. S6 (CH4 reduction of 9%) |  |  |  |  | 5.34E+00 | **75.9%** |  |  |  |  |  |  | 1.69E-03 | **37.8%** |  |  |  |  |  |  | 7.10E-01 | **99.9%** |  |  |
| 7. S7 (CH4 reduction of 10%) |  |  |  |  |  |  | 5.41E+00 | **77.0%** |  |  |  |  |  |  | 1.69E-03 | **37.8%** |  |  |  |  |  |  | 7.10E-01 | **99.9%** |
| 8. Slaughtering | 2.75E-02 | **0.4%** | 2.75E-02 | **0.4%** | 2.75E-02 | **0.4%** | 2.75E-02 | **0.4%** | 2.95E-05 | **0.7%** | 2.95E-05 | **0.7%** | 2.95E-05 | **0.7%** | 2.95E-05 | **0.7%** | 3.17E-09 | **0.0%** | 3.17E-09 | **0.0%** | 3.17E-09 | **0.0%** | 3.17E-09 | **0.0%** |
| **Total** | **5.81E+00** | **82.2%** | **5.79E+00** | **82.4%** | **7.03E+00** | **100.0%** | **6.11E+00** | **86.9%** | **1.73E-03** | **38.6%** | **1.87E-03** | **41.9%** | **4.47E-03** | **100.0%** | **2.47E-03** | **55.2%** | **7.11E-01** | **99.6%** | **7.14E-01** | **100.0%** | **7.10E-01** | **99.5%** | **7.10E-01** | **99.5%** |
|  | **Land use (Pt)** | | | | | | | | **Resource use, fossils (MJ)** | | | | | | | | **Water use (m3 depriv.)** | | | | | | | |
| **Life Cycle Stage** | **Baseline** |  | **S5** |  | **S6** |  | **S7** |  | **Baseline** |  | **S5** |  | **S6** |  | **S7** |  | **Baseline** |  | **S5** |  | **S6** |  | **S7** |  |
| 1A. AN Harvesting |  |  | 7.52E-03 | **0.0%** | 1.50E-02 | **0.0%** |  |  |  |  | 6.15E-02 | **0.1%** | 1.23E-01 | **0.3%** |  |  |  |  | 2.90E-05 | **0.0%** | 5.80E-05 | **0.0%** |  |  |
| 1B. LH Harvesting |  |  |  |  |  |  | 3.57E-01 | **0.0%** |  |  |  |  |  |  | 5.92E+00 | **12.6%** |  |  |  |  |  |  | 5.43E-03 | **0.3%** |
| 2A. Drying & Milling |  |  | 4.30E-02 | **0.0%** | 8.60E-02 | **0.0%** |  |  |  |  | 1.48E+00 | **3.2%** | 2.95E+00 | **6.3%** |  |  |  |  | 1.29E-02 | **0.7%** | 2.59E-02 | **1.3%** |  |  |
| 3A. Fermentation in Bioreactor |  |  |  |  | 0.00E+00 | **0.0%** |  |  |  |  |  |  | 1.62E+01 | **34.5%** |  |  |  |  |  |  | 9.97E-02 | **5.0%** |  |  |
| 3B. Alginate Production |  |  |  |  |  |  | 4.70E+00 | **0.6%** |  |  |  |  |  |  | 5.40E+00 | **11.5%** |  |  |  |  |  |  | 7.26E-01 | **36.6%** |
| 4A. Harter Drying |  |  |  |  | 0.00E+00 | **0.0%** |  |  |  |  |  |  | 2.11E+01 | **45.1%** |  |  |  |  |  |  | 1.30E-01 | **6.6%** |  |  |
| 4B. Pressing & Drying |  |  |  |  |  |  | 0.00E+00 | **0.0%** |  |  |  |  |  |  | 7.33E-02 | **0.2%** |  |  |  |  |  |  | 4.52E-04 | **0.0%** |
| 5. Packaging |  |  | 2.04E-02 | **0.0%** | 4.07E-02 | **0.0%** | 2.04E-02 | **0.0%** |  |  | 1.30E-01 | **0.3%** | 2.61E-01 | **0.6%** | 2.35E-04 | **0.0%** |  |  | 3.90E-03 | **0.2%** | 7.81E-03 | **0.4%** | 3.90E-03 | **0.2%** |
| 6. Transport |  |  | 6.36E-02 | **0.0%** | 1.27E-01 | **0.0%** |  |  |  |  | 1.33E-01 | **0.3%** | 2.66E-01 | **0.6%** |  | **0.1%** |  |  | 7.04E-04 | **0.0%** | 1.41E-03 | **0.1%** |  | **0.0%** |
| 6B. Shipping to Norwegian farm |  |  |  |  |  |  | 1.79E-02 | **0.0%** |  |  |  |  |  |  | 3.64E-02 | **0.1%** |  |  |  |  |  |  | 1.50E-04 | **0.0%** |
| 7. Baseline Sheep Farm | 7.43E+02 | **99.6%** |  |  |  |  |  |  | 5.77E+00 | **12.3%** |  |  |  |  |  |  | 1.26E+00 | **63.3%** |  |  |  |  |  |  |
| 7. S5 (CH4 reduction of 4%) |  |  | 7.42E+02 | **99.4%** |  |  |  |  |  |  | 5.72E+00 | **12.2%** |  |  |  |  |  |  | 1.25E+00 | **63.1%** |  |  |  |  |
| 7. S6 (CH4 reduction of 9%) |  |  |  |  | 7.42E+02 | **99.4%** |  |  |  |  |  |  | 5.72E+00 | **12.2%** |  |  |  |  |  |  | 1.25E+00 | **63.1%** |  |  |
| 7. S7 (CH4 reduction of 10%) |  |  |  |  |  |  | 7.41E+02 | **99.3%** |  |  |  |  |  |  | 5.71E+00 | **12.2%** |  |  |  |  |  |  | 1.25E+00 | **63.1%** |
| 8. Slaughtering | 0.00E+00 | **0.0%** | 0.00E+00 | **0.0%** | 0.00E+00 | **0.0%** | 0.00E+00 | **0.0%** | 2.34E-01 | **0.5%** | 2.34E-01 | **0.5%** | 2.34E-01 | **0.5%** | 2.34E-01 | **0.5%** | -3.56E-03 | **-0.2%** | -3.56E-03 | **-0.2%** | -3.56E-03 | **-0.2%** | -3.56E-03 | **-0.2%** |
| **Total** | **7.43E+02** | **99.6%** | **7.42E+02** | **99.4%** | **7.42E+02** | **99.4%** | **7.46E+02** | **100.0%** | **6.00E+00** | **12.8%** | **7.76E+00** | **16.6%** | **4.68E+01** | **100.0%** | **1.74E+01** | **37.1%** | **1.25E+00** | **63.1%** | **1.27E+00** | **63.8%** | **1.51E+00** | **76.3%** | **1.98E+00** | **100%** |
